# Infant nutrition affects the microbiota-gut-brain axis: Comparison of human milk vs. infant formula feeding in the piglet model

**DOI:** 10.3389/fnut.2022.976042

**Published:** 2022-09-21

**Authors:** Elise Charton, Alexandre Bourgeois, Amandine Bellanger, Yann Le-Gouar, Patrice Dahirel, Véronique Romé, Gwenaelle Randuineau, Armelle Cahu, Paul J. Moughan, Carlos A. Montoya, Sophie Blat, Didier Dupont, Amélie Deglaire, Isabelle Le Huërou-Luron

**Affiliations:** ^1^STLO, INRAE, Institut Agro, Rennes, France; ^2^Institut NuMeCan, INRAE, INSERM, Univ Rennes, Saint-Gilles, France; ^3^CHU Rennes, CIC-Inserm 1414, Rennes, France; ^4^Riddet Institute, Massey University, Palmerston North, New Zealand; ^5^Smart Foods and Bioproducts Innovation Centre of Excellence, AgResearch Limited, Palmerston North, New Zealand

**Keywords:** human milk, infant nutrition, intestinal immune system, intestinal physiology, microbiota, brain, hypothalamus

## Abstract

Early nutrition plays a dominant role in infant development and health. It is now understood that the infant diet impacts the gut microbiota and its relationship with gut function and brain development. However, its impact on the microbiota-gut-brain axis has not been studied in an integrative way. The objective here was to evaluate the effects of human milk (HM) or cow’s milk based infant formula (IF) on the relationships between gut microbiota and the collective host intestinal-brain axis. Eighteen 10-day-old Yucatan mini-piglets were fed with HM or IF. Intestinal and fecal microbiota composition, intestinal phenotypic parameters, and the expression of genes involved in several gut and brain functions were determined. Unidimensional analyses were performed, followed by multifactorial analyses to evaluate the relationships among all the variables across the microbiota-gut-brain axis. Compared to IF, HM decreased the α-diversity of colonic and fecal microbiota and modified their composition. Piglets fed HM had a significantly higher ileal and colonic paracellular permeability assessed by *ex vivo* analysis, a lower expression of genes encoding tight junction proteins, and a higher expression of genes encoding pro-inflammatory and anti-inflammatory immune activity. In addition, the expression of genes involved in endocrine function, tryptophan metabolism and nutrient transport was modified mostly in the colon. These diet-induced intestinal modifications were associated with changes in the brain tissue expression of genes encoding the blood-brain barrier, endocrine function and short chain fatty acid receptors, mostly in hypothalamic and striatal areas. The integrative approach underlined specific groups of bacteria (Veillonellaceae, Enterobacteriaceae, Lachnospiraceae, Rikenellaceae, and Prevotellaceae) associated with changes in the gut-brain axis. There is a clear influence of the infant diet, even over a short dietary intervention period, on establishment of the microbiota-gut-brain axis.

## Introduction

Human milk (HM) is assumed to meet the nutritional needs of infants, and to promote their optimal growth and development, including cognition, and favors beneficial bacteria related to health (1). Despite WHO recommendations (2), the exclusive breastfeeding rate is still low and world-wide reaches only 44% of infants aged 0 to 5-months (3). Infant formulas (IFs) have a close nutritional composition to HM. They are not identical, however, differing compositionally and structurally. Consequently, IF may not provide all of the physiological benefits associated with HM. For instance, breastfeeding reduces the risk of developing diarrhea or otitis within the first years of life (4, 5) and modulates the development of the intestinal immune system (6–9). Its effect on intestinal barrier function is still debated (10–13). Breastfeeding also benefits on brain development, resulting in improved language and motor function and learning abilities (14–16).

HM contains a myriad of bioactive substances including proteins and lipids, and acts as a prebiotic and probiotic due to its oligosaccharides and microbial composition that are not yet mimicked in IFs (1). IFs are mostly formulated with bovine proteins, which differ from HM proteins. Consequently, to cover the infant amino acid requirements, IF must contain a higher protein content than HM (on average: 1.3 vs. 0.8–1.2 g/100 mL, respectively) (17). This is partly due to the limiting content of tryptophan in bovine whey proteins. Tryptophan has been particularly studied over the past decades in relation to both intestinal and brain functions (18–22). While HM and IF lipid concentrations are usually similar (3.4 g/100 mL), lipid composition and structure differ due to their origin, with plant lipids mostly used in IFs (23), and due to IF homogenization that transforms the large Milk Fat Globule into smaller lipid droplets (24).

These differences between HM and IF could explain reported differences in intestinal bacterial ecosystems and health outcomes. The fecal microbiota of breastfed infants has a low α-diversity and is characterized by a low relative abundance of Firmicutes at 3–6 months of age (25, 26). Proteobacteria, scarcely abundant in the first weeks of life, persist as a low abundant phylum throughout the first 2.5 years of life (25–27). *Bifidobacterium* is highly abundant in the early postnatal days and *Bacteroides* increases over the first month of life of breastfed infants (28). In contrast, a higher α-diversity is commonly reported in IF-fed compared to HM-fed infants up to 6 months of age (26, 27, 29). The most abundant genera also differ with a higher abundance of *Clostridium*, *Enterococcus*, and *Klebsiella* in 1- and 4-week-old IF-fed infants, unlike in HM-fed infants who have a higher abundance of *Bifidobacterium*, *Bacteroides*, and *Staphylococcus* (6). The major role of the microbiota on gut immune system and barrier maturation (30–33) as well as on brain development has been widely studied (34–37). Many studies have shown that the gut microbiota influences neuro-development during the first years of life notably by reducing the risk of developing some neuropsychiatric or neurodevelopmental diseases (35, 38–41). In 3 year-old term-children, negative correlations between dominance of Clostridiales and communication, personal and social skills, and between high abundance of *Bacteroides*, low abundance of *Escherichia/Shigella* and *Bifidobacterium* with fine motor skills have been observed (42).

During the past few years, many studies have analyzed the impact of the infant diet on the microbiota and its relationship with gut function or brain development. To date, however, no integrative study has evaluated collectively the modulation of the microbiota-gut-brain axis by the infant diet. This is the aim of the present paper, comparing HM vs. IF, using Yucatan piglets as a human infant model. The suckled piglet is well established as a suitable animal model for human infants (43–45) and the Yucatan miniature pig has the advantage of needing less amount of milk. Intestinal microbiota composition, intestinal phenotypic parameters and intestinal and brain gene expression patterns were determined before proceeding with multifactorial and correlation analyses to evaluate the relationships among all the variables across the microbiota-gut-brain axis.

## Materials and methods

### Human milk sample collection

The protocol for HM collection for the present pre-clinical study was approved by the Institutional Review Board of South Mediterranean V (no19.12.12.65653). Two types of HM pools were used. Regarding the first pool, HM samples were obtained frozen from the donor milk bank of the Rennes University Hospital Centre. HM samples were heat-treated following the Holder pasteurization (62.5°C, 30 min). Milk from 22 donating mothers (range of women’s lactation period: 0.30 – 5.61 months post-partum) were analyzed for crude protein, tryptophan and fat content and the heat-treated HM was pooled. This pooled HM had a similar chemical composition to that of the IF (Table 1). The heat-treated HM pool was stored at −20°C and thawed overnight before being given to the piglets as detailed below. For the second HM pool, fresh HM samples were collected from 50 healthy mothers (range of women’s lactation period: 1.76 – 1.96 months post-delivery), pooled and stored at 4°C until use the day after collection.

**TABLE 1:** Protein, tryptophan, lipid, and HM oligosaccharide content of pasteurized human milk^§^ (PHM) and the infant formula (IF) (mean ± SEM).

g/100 mL	Pasteurized human milk (PHM)^§^	Infant formula (IF)	*P*-value
Crude protein^¤^	1.67 ± 0.05	1.44 ± 0.02	0.014
True protein^†^	1.28 ± 0.03	1.37 ± 0.02	0.085
Tryptophan	0.028 ± 0.001	0.026 ± 0.001	0.339
Lipids	3.21 ± 0.15	3.15 ± 0.06	0.714
HM oligosaccharides^‡^	0.87 ± 0.01	−	−

^§^Pool of 22 anonymous donations of human milk heat treated by Holder pasteurization range of women’s lactation period: 0.3 – 5.61 months, gestational age range: 23–38 weeks.

¤Crude protein = total nitrogen * 6.38.

^†^True protein = (total nitrogen – non-protein nitrogen) * 6.38.

^‡^Mean concentration (g/100 mL) of the top 20 oligosaccharides found in the highest concentrations: 2′FL: 0.163 ± 0.010; 3′FL: 0.090 ± 0.004; 6′SL/LNT: 0.086 ± 0.002; LNDFH1: 0.051 ± 0.006; MFLNH 3: 0.039 ± 0.001; LNFP2: 0.037 ± 0.003; LNFP1: 0.035 ± 0.007; 2HexNAc4Hex3Fuc: 0.029 ± 0.001; LDFT/DFL: 0.027 ± 0.001; DFLNH: 0.025 ± 0.002; Neu5Ac4Hex2HexNAcFuc: 0.021 ± 0.001; LNFP3: 0.020 ± 0.001; DFLNH2: 0.016 ± 0.001; 3′SL: 0.015 ± 0.0004; 6′SLNT: 0.014 ± 0.01; LnNT: 0.014 ± 0.001; DFLNH a: 0.013 ± 0.001; LNH/LnNH: 0.010 ± 0.0003; DFLNH3: 0.010 ± 0.01.

### Animal study and diets

The study was designed and conducted in compliance with the current ethical standards of the European and French guidelines. The ethics committees of CREEA (Rennes Committee of Ethics in Animal Experimentation) and of France’s Ministry of Higher Education and Research approved the protocol (authorization #2020020610329770). Animals were observed daily throughout the experimental protocol to ensure their welfare and they did not receive medication or antibiotic treatment.

Eighteen 10-day-old healthy Yucatan piglets (10 female and 8 male) were housed individually in stainless steel metabolic cages. Room temperature was maintained at 26 ± 2°C with a 15:9 h light/dark cycle. Piglets received 345 mL of diet/kg BW/day partitioned in 10 meals distributed from 7:30 to 22:00. The diet was given via a drinking trough. During the study, piglets were weighed every 3 days to adjust their food ration. Piglets were fed with an adaptation diet for 8 ± 2 days (adaptation period), based on a full fat bovine milk powder (Euroserum, Sodiaal, France, Supplementary Table 1) enriched with vitamins and minerals (0.5% powder, Piglet premix Step 1, Mg2Mix, France) and rehydrated with ultrapure water (14.5 g/100 mL diet). Then, piglets were randomly assigned to one of the two dietary groups and received their specific diets for 6 days (experimental period). Allocation to each diet was balanced between groups according to BW at 10 days of age, litter origin and sex. The IF piglet group was fed with a standard cow’s milk based IF, produced within our laboratory such as previously described (46). IF powder was rehydrated with ultrapure water daily (11.5 g/100 mL diet) and stored at 4°C until feeding. Due to the limited access to fresh HM, the HM piglet group was fed with the pasteurized HM pool during the first 5 days and with the fresh HM pool on the last day of the experiment. Ytterbium and Cobalt-EDTA were added to IF and HM diets at a level of 0.3 g/100 g dry matter to allow determination of digestibility values (data not shown). All diets were supplemented with liquid vanilla (0.3 g/100 mL diet) to encourage intake during both adaptation and experimental (IF or HM) periods. Dietary intake was recorded at each meal. The experiment was undertaken over 3 independent blocks.

### Sample collection

During the adaptation period, feces were collected over the last 2 days and stored at −80°C for microbiota analysis. On the last day of the experiment, animals received 6 meals distributed every hour and were euthanized 30 min after the last meal by electrical stunning immediately followed by exsanguination. Blood was collected in tubes containing K^2^-EDTA plus an anti-dipeptidyl-peptidase-IV (anti-DPP-IV, 10 μL/mL of blood) for GLP1 analysis (Millipore, Billerica, MA, United States). After centrifugation (10 min, 2500 g, 4°C), plasma samples were stored at −80°C. Ileal digesta and tissues were collected over 60 cm anterior to the ileocecal junction and colonic digesta and tissues were collected from the first third of the colon. Ileal and colonic digesta and feces were immediately frozen in liquid nitrogen and stored at −80°C for microbiota analysis. The ileum and proximal colon were dissected and rinsed with cold phosphate buffered saline (PBS). Ten-cm segments were kept in ice-cold Dulbecco’s Minimum Essential Medium (Gibco, Thermo Fisher) for immediate Ussing chamber analysis. About 100 mg of ileal and proximal colonic tissues were sampled and kept in an RNA later solution for 24 h at 4°C and stored at −20°C for RNA extraction and gene expression analysis. Adjacent segments (10-cm) were fixed in 4% paraformaldehyde for 48 h until further dehydration in ethanol and embedding in paraffin, for morphometry analysis and GLP1, chromogranin and goblet cell counting. Finally, ileal and colonic tissues (1 cm) were immediately frozen in liquid nitrogen and stored at −80°C until GLP1 extraction and assay. Two pieces of liver (100 mg each) were collected, immediately frozen in liquid nitrogen and stored at −80°C for RNA extraction. Immediately after euthanasia, the brain was extracted and four regions of interest (hypothalamus, striatum, prefrontal cortex, and hippocampus from the right hemisphere) were sampled. Brain samples were immediately frozen in liquid nitrogen and stored at −80°C for RNA extraction and gene expression analysis.

### Diet biochemical analysis

#### Crude and true protein content

The total nitrogen content was measured in duplicate using the Dumas method (ISO 14891:2008) on a LECO FP 828 analyzer after calibration using EDTA. A protein factor conversion of 6.38 was used to determine the crude protein content. Total N was corrected for non-protein nitrogen and then multiplied by the protein factor conversion of 6.38 to determine the true protein content.

#### Tryptophan content

The tryptophan content was determined based on the method of the European Commission (47) and the ISO 13904:2016 (48). HM and rehydrated IF were heated up to 35°C. Each diet sample, containing at least 20 mg of crude protein, was inserted in a 13 mL screw cap Teflon tube and diluted with 2 mL of ultrapure water containing 1.05 g of octa-hydrated barium hydroxide before a 16 h hydrolysis in autoclave at 110°C. After hydrolysis, 5-methyl-tryptophan was added as an internal standard at a level of 1.0 – 3.5 mg/L before mixing and cooling down on ice for 15 min. After centrifugation at 4,000 *g* for 2 min at 8°C, supernatants were diluted in acid acetic 10%, filtered through a 0.2 μm pore-size membrane (Chromafil Xtra Filter, 13 mm, PTFE) prior to HPLC analysis. Samples were analyzed by RP-HPLC using a Symmetry C18 (5 μm) column (2.1 mm × 150 mm, WATERS) with an isocratic elution (95% Sodium acetate 0.4% pH = 4.5; 5% Pure Acetonitrile) at 0.25 mL/min. An L-tryptophan standard with a range of concentration from 0 to 10 mg/L, corrected by the internal 5-methyl-tryptophan standard (3.57 mg/L), was used for tryptophan content determination. The detection was made by fluorimetry, using excitation and emission wavelengths of 280 and 346 nm, respectively. Tryptophan content was corrected for losses occurring during basic hydrolysis, estimated after performing multiple hydrolysis of the samples.

#### Total lipid content

Diet samples (500 μL) were precisely weighed into a 15 mL screw cap Teflon tube before addition of 10 mL of Folch reagent (chloroform/methanol, 2/1, v/v). After 1 h of rotative agitation, 2 mL of KCL 0.8% was added. After centrifugation (5 min, 450 g, 20°C), the solvent phases were rinsed twice with a chloroform/methanol/KCl 0.8% solution (3/48/47, v/v/v) and filtered (Whatman filter paper, 1PS). The filtrate solvent was evaporated under a nitrogen flow in a 40°C water bath and the remaining total lipids were precisely weighed.

### Microbiota analysis

Extraction of total bacterial DNA from feces, colonic and ileal digesta was performed as described in the instruction guide of the Quick-DNA Fecal/Soil Microbe Miniprep Kit (ZYMO Research). After extraction, bacterial DNA extracts were sequenced for 16s rRNA using Illumina Miseq protocol (49) (INRAE GenoToul platform, Toulouse).

### Morphometry and immunohistochemical analyses

Morphometric analysis was performed after alcian blue and periodic acid Schiff staining on 7 μm sections of formalin-fixed, paraffin-embedded ileal and proximal colonic tissues. Sections were examined under a light microscope (Nikon Eclipse E400, Nikon Instruments, France) using image analysis software (NIS-Elements AR 3.0, Nikon Instruments) as described by Le Bourgot et al. (50). Villus and crypt length, width and surface area were measured in at least 15–20 crypt-villus units per piglet. Goblet cells were also counted per villus and per crypt using the same staining condition. Immunohistochemical analysis of ileum and colon sections was processed as previously described (51) to determine the number of enteroendocrine (chromogranin A-labeled) cells and GLP1-containing cells per area of mucosa.

### Plasma and tissue GLP1 concentration

GLP1 content was extracted from ileal and proximal colonic tissue by homogenization of 1 g of tissue in 5 mL of ethanol acid solution (1% HCl 12 M, 74% absolute ethanol, 25% H_2_O) (Polytron 3100, Kinematica, 24,000 rpm, 2 s × 20 s). After 24 h at 4°C, samples were centrifuged (20 min, 2,000 *g*, 4°C) and supernatants diluted (1:1000 and 1:250 for ileum and colon, respectively). Intestinal and plasma GLP1 concentration was measured using a GLP1 active ELISA kit (Millipore), according to the manufacturer instructions.

### *Ex vivo* permeability measurement

Ileal and colonic permeability measurements were performed using Ussing chambers (Physiological Instruments, San Diego, CA, United States). Permeability was determined using tracer molecules Na-FITC for paracellular permeability and horseradish peroxidase (HRP) for transcellular permeability. The tracer molecules were added into the apical compartment and those transferred through the epithelium were analyzed in the serosal compartment. Concentration of Na-FITC in the samples collected at 30-min intervals for 120 min from the serosal buffer was measured by fluorimetry (fluorimeter LB940 Mithras, Berthold Technologies, Thoiry, France), while concentration of HRP was determined using spectrophotometry (Multiskan spectrum, Thermo Labsystem, Midland, Canada) after enzymatic reaction using *o*-dianisidine as substrate (Sigma-Aldrich). Mucosal-to-serosal fluxes were then calculated and expressed as ng/cm^2^/h (52).

### Gene expression analysis

Total RNA extraction from intestinal tissues and liver was performed using the “NucleoSpin^®^ RNA” kit (Macherey Nagel) or using the RNeasy Plus Universal Mini kit (Qiagen) for brain tissues. Extracted RNA were quantified using a Denovix spectrophotometer. RNA quality and integrity controls were performed with Agilent RNA 6000 Nano kit utilizing an Agilent 2100 Bioanalyzer (Agilent Technologies France, Massy, France) and by calculation of the RIN (RNA Integrity Number). All RIN had good quality. Reverse transcription was then performed on 2 μg of extracted RNAs with the High Capacity Complementary DNA Reverse Transcription Kit, as previously described (51).

The gene expression analysis was carried out in brain and intestine using the Smartchip Real time PCR through syber green technology using the Wafergen Smartchip cycler and Smartchip Multisample Nanodipenser (Biogenouest Genomics and the EcogenO core facility of Rennes, France). Two dedicated porcine smartchips were designed in-house to specifically investigate targeted gene expression in the intestine (‘Porcine Gut Smartchip’) and in the brain (‘Porcine Brain Smartchip’). Forthe ‘Porcine Gut Smartchip,’ the expression of 106 genes targeted on specific intestinal functions and of 12 housekeeping genes was analyzed in ileum and colon (Supplementary Table 2). The porcine Gut Smartchip was focused on genes related to immune system, barrier function, endocrine function, digestion/nutrient carriers, and tryptophan pathways. For the ‘Porcine Brain Smartchip,’ the expression of 63 targeted genes and of 10 housekeeping genes was analyzed in the 4 areas of interest (Supplementary Table 3). The porcine Brain Smartchip was focused on genes related to barrier function, immune system, endocrine function, neurosynaptogenesis function, neurotransmitters, nutrient carriers and tryptophan pathways. The steadiest housekeeping genes, selected with *Genorm software*, were PPIA, RPL4, HPTR1, and POLR2G for the intestine (ileum and colon), and ACTB, ALDOA, B2M, HPTR1, PGK1, RPL4, and YWHAZ for the brain areas. In liver, the expression of 5 genes of interest (IDO, KMO, KYAT, TDO, and TPH1) were also analyzed using RT-qPCR technology of the PCR Step One Plus (Applied Biosystems). Relative expressions of the target genes were determined using the 2^–ΔΔ*Ct*^ method to compare the IF group to the HM group. Full dataset is available on an online dataverse https://doi.org/10.57745/5FHAYQ.

### Statistical analysis

Data are presented as mean ± standard error of the mean (SEM).

#### Microbiota data analysis

Raw sequences that were obtained from microbiota analysis were analyzed using the bioinformatic pipeline FROGS (Find Rapidly OTU with Galaxy Solution) software (53).

The descriptive analysis of the structure (α and β diversity) of microbiota was conducted with the Phyloseq function (EdgeR package, Bioconductor). The α-diversity indices used were Observed species and Chao1 representing the bacterial richness, and Shannon and InvSimpson representing the bacterial equitability. Significant differences between dietary groups were assessed using ANOVA (aov function). Phylogenetic β diversity was studied using the Unifrac distance and group differences was evaluated with principal co-ordinate analysis (PCoA) and permutational multivariate analysis (PERMANOVA) of variance using distance matrices. Differences in phyla, families and genera were assessed with pairwise comparisons, after aggregation at the desired taxonomic rank (phyloseq’ tax_glom function) by using EdgeR package (Bioconductor). Multiple testing corrections (False Discovery Rate) were used to avoid false positives (significance threshold = 0.05).

#### Unidimensional analysis

All statistical analyses were performed using the R software, version 3.6.2 (54).

A linear model was used to test the statistical significance of the dietary treatment on milk lipid, protein and tryptophan dietary contents. A linear mixed model was used to test the impact of diet, time, block, sex and their 2-by-2 interactions on piglet growth and food intake. Piglet was considered as a random effect, and other parameters were fixed effects. A linear model was used to test the impact of diet, block and sex on gene expression, permeability measurement and morphometry data where parameters were fixed effects. For both models, when the sex or block effects were non-significant (*p* > 0.1), these factors were removed from the linear model. The normality distribution and the homoscedasticity of the residuals of each linear model were tested using Shapiro–Wilk and Levene’s tests, respectively (55). Models were considered as acceptable for *p* > 0.05. When the raw data did not fulfill these model assumptions, a natural logarithmic transformation of the data was performed prior to running the linear models. If the assumptions were still not satisfied, data were tested with a non-parametric Wilcoxon test. Differences were considered as statistically significant for *p* < 0.05 and a trend for difference at 0.05 < *p* < 0.1.

#### Data correlation

Pearson correlation coefficients were determined between gut microbiota variables (phylum, family and genus abundances in ileum and colon), intestinal variables (morphometry, permeability, gene expression, goblet cell counting, GLP1 content, and cell numbers), and brain variables (gene expression over the four brain areas). Correlations were considered significant when *p* < 0.05 and | *r*| > 0.7. Full dataset is available on an online dataverse https://doi.org/10.57745/5FHAYQ.

#### Multidimensional analysis

The Multi-factor analysis [FACTOMINE R package (56, 57)] was performed on all the data including microbiota, intestinal (ileum and colon) and brain (four areas) variables to integrate the global dietary effect on the microbiota-gut-brain axis. Variables were divided into three groups: (1) microbiota (ileum and colon) variables; (2) intestinal (ileum and colon) variables; (3) brain variables.

## Results

### Diet composition

The dietary crude protein content was significantly higher in HM than in IF (Table 1) while there was no statistically significant difference for the content in true protein, tryptophan or lipid between diets. HM contained 0.87 ± 0.01 g/100 mL oligosaccharides while no oligosaccharides were added to the IF (Table 1).

### Piglet growth and food intake

There was no significant diet effect on piglet growth, with an average daily weight gain of 54 ± 5 g/day. The dietary intake was significantly higher for HM on the 2nd day of the experimental period, while no further differences in intake between the HM and IF groups was observed. HM and IF daily intakes were on average 270 ± 10 and 240 ± 10 g/kg BW/day, respectively.

### Intestinal microbiota

**FIGURE 1:**
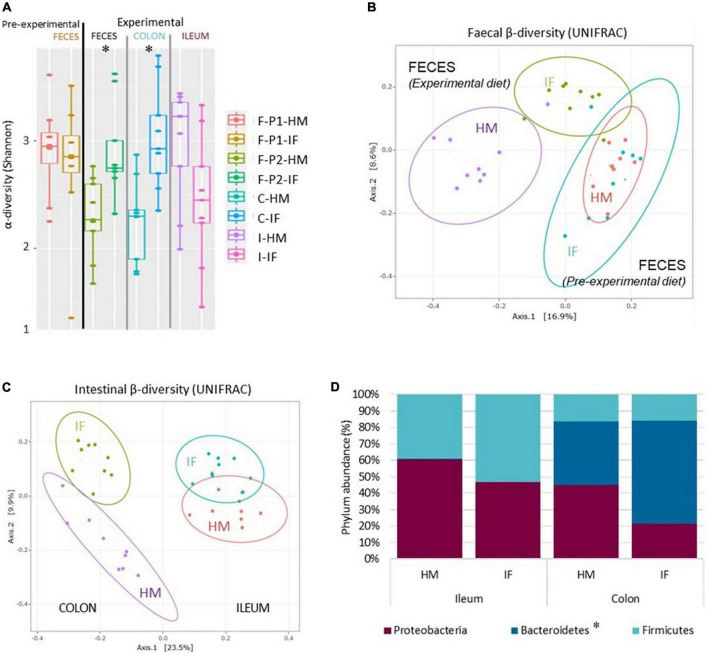
Microbiota composition and diversity. **(A)** Microbial α-diversity (Standardized Shannon Index) in feces (F-), and colonic (C-) and ileal (I-) digesta of HM- and IF-fed piglets during the adaptation period (P1, bovine milk) and the experimental (HM or IF) period (P2). **(B)** Microbial fecal β-diversity (Unifrac index) of HM- and IF-fed piglets during the adaptation (blue and orange ovals) and the experimental (purple and green ovals) periods. **(C)** Microbial ileal and colonic β-diversity (Unifrac Index) during the experimental period. **(D)** Most abundant phyla in ileal and colonic digesta of HM- and IF-fed piglet (abundance > 0.05%), *phylum abundance significantly different in colonic digesta of HM-fed piglets compared to IF-fed piglets, *P* < 0.05.

Before the start of the experimental diet period, the fecal α-diversity was similar between piglets (Figure 1A), while feeding HM vs. IF induced changes in fecal α-diversity (Figure 1A). The fecal Shannon index of HM-fed piglets was significantly reduced after a 6-day dietary intervention, illustrating a significantly lower richness and equitability of HM-induced bacterial ecosystem compared to the IF-induced one. Significantly lower Chao1 (data not shown) and Shannon α-diversity indices were also observed in the colonic digesta for HM-fed piglets, but no difference was observed in ileal digesta (Figure 1A).

The fecal β-diversity, represented by the Unifrac index, confirmed that there was no difference before the start of the experimental period, whereas feeding piglets with HM or IF induced bacterial ecosystem changes in feces (Figure 1B) and colonic digesta (Figure 1C). In addition, the β-diversity analysis showed differences between ileal and colonic microbiota (Figure 1C), with a less apparent impact of the diet on the ileal microbiota.

The microbial composition was diet-dependent in the colon (Figure 1D and Table 2.1) and in the feces (Table 2.1), but not in the ileum (Figure 1D). At the phylum level, the relative abundances of Bacteroidetes, Deferribacteres and Fusobacteria were lower in colon and feces (Table 2.1), but not different in the ileum, of HM-fed piglets compared to IF-fed piglets (Figure 1D). At the family level, 4, 15, and 13 taxa were differentially abundant in ileum, colon and feces of HM- vs. IF-fed piglets, respectively (Table 2.2). They corresponded to 15, 24, and 28 differential genus abundance in ileum, colon and feces, respectively (Tables 3.1, 3.2). In the ileum, all differential genera of Proteobacteria and Bacteroidetes were more abundant in HM- than in IF-fed piglets, while the Firmicutes genera were less abundant, except for *Lactobacillus*. In the colon, genera of Firmicutes and Proteobacteria represented the majority (50 and 29%, respectively) of the differentially abundant taxa. Similarly, the differential abundant genera in feces corresponded to genera of Firmicutes and Proteobacteria (39 and 32%, respectively). Fecal *Bifidobacterium* (Actinobacteria) was more abundant in HM-fed piglets than in IF-fed piglets.

**TABLE 2.1:** Dietary impact on relative abundance of bacterial phyla in colon and feces of HM- and IF-fed piglets.

Comparison	Phylum	*p*-adjusted	Log_2_(Fold Change)
HM vs. IF (colon)	Bacteroidetes	0.006	−2.18
	Deferribacteres	0.043	−6.58
	Fusobacteria	0.006	−3.18
HM vs. IF (feces)	Actinobacteria	0.013	2.30
	Bacteroidetes	0.001	−2.20
	Deferribacteres	0.001	−6.60
	Fusobacteria	0.004	−3.20

Log_2_FoldChange, log_2_(abundance HM/abundance IF). The Foldchange indicates the differential abundance of phyla in HM group compared to IF group. For example: *Bacteroidetes* abundance is lower in HM colon compared to IF one.

**TABLE 2.2:** Dietary impact on relative abundance of bacterial families in ileum, colon and feces of HM- and IF-fed piglets.

Comparison	Phylum	Family	*p*-adjusted	Log_2_ (FoldChange)
HM vs. IF (ileum)	Firmicutes	Erysipelotrichaceae	<0.001	−8.63
	Proteobacteria	Aeromonadaceae	<0.001	13.84
		Pseudomonadaceae	<0.001	8.45
		Xanthomonadaceae	0.005	4.63
HM vs. IF (colon)	Actinobacteria	Coriobacteriaceae	<0.001	4.29
		Eggerthellaceae	0.030	−3.32
	Bacteroidetes	Marinifilaceae	<0.001	−5.34
		Muribaculaceae	<0.001	−4.94
		Prevotellaceae	<0.001	−5.66
		Rikenellaceae	<0.001	−3.83
	Epsilonbacteraeota	Campylobacteraceae	0.006	7.16
	Firmicutes	Erysipelotrichaceae	0.001	−3.31
		Peptococcaceae	<0.001	−20.57
		Veillonellaceae	<0.001	6.05
	Proteobacteria	Aeromonadaceae	0.026	5.91
		Desulfovibrionaceae	0.006	2.27
		Enterobacteriaceae	<0.001	3.53
		Pasteurellaceae	0.012	−1.96
		Unclassified_Bradymonadales order	0.005	−9.94
HM vs. IF (feces)	Actinobacteria	Eggerthellaceae	0.009	−4.01
	Bacteroidetes	Marinifilaceae	<0.001	−6.9
		Muribaculaceae	<0.001	−6.25
		Prevotellaceae	0.013	−3.3
		Rikenellaceae	<0.001	−4.63
	Deferribacteres	Deferribacteraceae	0.012	−4.95
	Firmicutes	Family XIII	<0.001	−5.92
		Peptococcaceae	0.002	−7.74
		Veillonellaceae	<0.001	4.45
	Proteobacteria	Aeromonadaceae	0.007	3.98
		Enterobacteriaceae	<0.001	3.37
		Nitrosomonadaceae	<0.001	−5.39
		Unclassified_Bradymonadales order	<0.001	−27.1

HM, human milk; IF, infant formula. The Foldchange indicates the differential abundance of families in HM group compared to IF group. For example: Erysipelotrichaceae abundance is lower in HM ileum compared to IF.

**TABLE 3.1:** Dietary impact on relative abundance of bacterial genera in the ileum and colon of HM- and IF-fed piglets.

Comparison	Phylum	Family	Genus	*p*-adjusted	Log_2_ (FoldChange)
**HM vs. IF (ileum)**	Bacteroidetes	Muribaculaceae	*Muribaculum*	<0.001	17.24
		Prevotellaceae	*Prevotella*	0.047	5.28
			*Prevotellaceae NK3B31 group*	0.026	5.40
	Firmicutes	Erysipelotrichaceae	*Turicibacter*	<0.001	−8.04
		Lachnospiraceae	*Blautia*	0.047	−3.47
		Lactobacillaceae	*Lactobacillus*	<0.001	2.27
	Proteobacteria	Aeromonadaceae	*Aeromonas*	<0.001	14.29
		Burkholderiaceae	*Sutterella*	0.011	4.11
		Enterobacteriaceae	*Kluyvera*	0.002	8.00
			*Enterobacter*	<0.001	10.92
			*Raoultella*	<0.001	42.77
			*Salmonella*	<0.001	12.27
		Moraxellaceae	*Acinetobacter*	<0.001	6.18
		Pseudomonadaceae	*Pseudomonas*	<0.001	10.94
		Xanthomonadaceae	*Stenotrophomonas*	<0.001	9.69
**HM vs. IF (colon)**	Bacteroidetes	Marinifilaceae	*Butyricimonas*	<0.001	−6.87
		Muribaculaceae	*Muribaculum*	<0.001	14.63
		Prevotellaceae	*Alloprevotella*	<0.001	−9.49
		Rikenellaceae	*dgA-11 gut group*	0.015	−6.71
			*Rikenellaceae RC9 gut group*	<0.001	−5.77
	Deferribacteres	Deferribacteraceae	*Mucispirillum*	0.041	−4.40
	Firmicutes	Erysipelotrichaceae	*Catenisphaera*	<0.001	−5.79
			*Sharpea*	0.005	8.35
			*Turicibacter*	0.009	−8.53
		Family XIII	*Family XIII AD3011 group*	0.013	−6.33
		Lachnospiraceae	*Blautia*	0.001	−4.68
		Ruminococcaceae	*[Eubacterium] coprostanoligenes group*	<0.001	−6.81
			*Ruminiclostridium 9*	0.031	−4.77
			*Ruminococcaceae UCG-004*	0.002	−4.41
			*Subdoligranulum*	<0.001	−5.68
		Veillonellaceae	*Megasphaera*	<0.001	7.12
			*Mitsuokella*	0.002	11.72
			*Veillonella*	<0.001	8.32
	Proteobacteria	Aeromonadaceae	*Aeromonas*	<0.001	17.31
		Desulfovibrionaceae	*Bilophila*	<0.001	−3.77
			*Unknown genus*	0.007	−4.79
		Enterobacteriaceae	*Escherichia-Shigella*	<0.001	3.23
			*Enterobacter*	0.002	4.13
			*Salmonella*	<0.001	11.85
		Nitrosomonadaceae	*GOUTA6*	0.007	−5.17

HM, human milk; IF, infant formula.

**TABLE 3.2:** Dietary impact on differential relative abundance of bacterial genera in feces of HM- and IF-fed piglets.

Comparison	Phylum	Family	Genus	*p*-adjusted	Log_2_ (FoldChange)
**HM vs. IF (feces)**	Actinobacteria	Bifidobacteriaceae	*Bifidobacterium*	0.034	3.56
		Coriobacteriaceae	*Collinsella*	<0.001	4.38
	Bacteroidetes	Bacteroidaceae	*Bacteroides*	0.034	2.09
		Marinifilaceae	*Butyricimonas*	0.002	−5.55
		Prevotellaceae	*Prevotella 2*	<0.001	−5.94
			*Alloprevotella*	0.033	−8.49
		Rikenellaceae	*Rikenellaceae RC9 gut group*	<0.001	−5.31
	Epsilonbacteraeota	Campylobacteraceae	*Campylobacter*	0.005	8.02
	Firmicutes	Erysipelotrichaceae	*Turicibacter*	<0.001	−6.4
			*Sharpea*	0.011	6.89
		Lachnospiraceae	*Blautia*	<0.001	−4.91
			*Lachnoclostridium*	0.010	2.82
		Lactobacillaceae	*Lactobacillus*	0.026	1.55
		Ruminococcaceae	*Subdoligranulum*	0.037	−4.81
			*[Eubacterium] coprostanoligenes group*	<0.001	−4.49
			*UBA1819*	0.008	6.32
		Veillonellaceae	*Anaerovibrio*	0.007	6.35
			*Megasphaera*	<0.001	6.3
			*Veillonella*	<0.001	7.76
	Proteobacteria	Aeromonadaceae	*Aeromonas*	0.018	7.18
		Desulfovibrionaceae	*Bilophila*	0.008	−3.16
			*Desulfovibrio*	0.008	2.6
			*Mailhella*	0.043	−3.73
		Enterobacteriaceae	*Enterobacter*	<0.001	5.24
			*Kluyvera*	0.014	6.22
			*Raoultella*	<0.001	36.94
			*Salmonella*	<0.001	14.79
		Moraxellaceae	*Acinetobacter*	0.022	3.41

HM, human milk; IF, infant formula.

Despite these different microbial profiles, fecal and colonic short chain fatty acid (SCFA) concentrations were not significantly different between HM- and IF-fed piglets with the highest concentrations being found for acetate and propionate (Supplementary Table 4). Total SCFA content was significantly higher in colon than in feces.

### Morphometry, goblet cell number, and epithelial barrier permeability

The dietary impact on ileal and colonic villi and crypts was moderate. The ileal crypt length was reduced by 19% in HM- compared to IF-fed piglets (Table 4). There was no diet effect on the goblet cell number, with mean values of 22 ± 2 goblet cells per villus and 16 ± 0.5 goblet cells per crypt in ileum and 38 ± 2 goblet cells per crypt in colon, in agreement with unchanged expression of genes involved in mucosal defense (BD2, Lyz, MUC1, and MUC2) and proliferation (PCNA) (data not shown).

**TABLE 4:** Ileal and colonic tissue characteristics.

			Human milk	Infant formula	*P*-value
Morphometry	Ileum	Villous area (μm^2^)	19442.9 ± 1649.1	18468.9 ± 2185.9	0.52
		Villous length (μm)	221.4 ± 18.7	217.2 ± 19.8	0.83
		Villous width (μm)	96.7 ± 2.5	94.0 ± 4.8	0.23
		Crypt area (μm^2^)	4294.8 ± 474.7	5175.0 ± 481.3	0.21
		Crypt length (μm)	111.4 ± 8.4	137.6 ± 8.9	0.05
		Crypt width (μm)	46.2 ± 2.3	43.3 ± 2.4	0.39
	Colon	Crypt area (μm^2^)	16416.9 ± 1215.6	16964.5 ± 1891.3	0.74
		Crypt length (μm)	270.6 ± 13.3	292.9 ± 29.5	0.61
		Crypt width (μm)	67.4 ± 3.4	65.5 ± 2.1	0.61
Permeability^†^	Ileum	Na-FITC permeability (ng/cm^2^/h)	42.8 ± 9.8	25.0 ± 3.0	0.03
		HRP permeability (ng/cm^2^/h)	61.3 ± 13.3	33.3 ± 6.8	0.07
	Colon	Na-FITC permeability (ng/cm^2^/h)	57.7 ± 5.3	42.3 ± 3.8	0.03
		HRP permeability (ng/cm^2^/h)	72.0 ± 22.0	60.3 ± 12.7	0.73
Goblet cells	Ileum	Mucin cell number per villous	23.3 ± 2.4	20.7 ± 2.7	0.76
		Mucin cell number per crypt	15.3 ± 0.7	16.3 ± 0.8	0.98
	Colon	Mucin cell number per crypt	37.4 ± 1.0	39.0 ± 3.9	0.70
Endocrine function	Ileum	GLP1 content (pM/g tissue)	127.9 ± 20.0	256.5 ± 24.1	<0.01
		GLP1 cell number per mm^2^	71.0 ± 3.9	65.4 ± 3.6	0.40
		Chrg A cell number per mm^2^	144.4 ± 9.1	147.3 ± 8.8	0.63
		Ratio GLP1/Chrg A cells per mm^2^ (%)	49.8 ± 2.1	45.0 ± 2.1	0.07
	Colon	GLP1 content (pM/g tissue)	105.2 ± 15.2	122.8 ± 14.2	0.26
		GLP1 cell number per mm^2^	18.8 ± 2.1	18.6 ± 1.6	0.59
		Chrg A cell number per mm^2^	49.2 ± 6.3	52.8 ± 4.4	0.66
		Ratio GLP1/Chrg A cells per mm^2^ (%)	39.0 ± 1.3	35.7 ± 2.0	0.08
	Plasma	GLP1 content (pM/g tissue)	26.8 ± 2.7	24.8 ± 3.8	0.68

Mean ± SEM, *n* = 9 per group, except for permeability

^†^ *n_HM_* = 6 piglets and *n_IF_* = 7 piglets; HM, human milk; IF, infant formula, Chrg, chromogranin.

The ileal and colonic paracellular (Na-FITC passage) permeability was significantly higher in HM-fed than in IF-fed piglets (Table 4). Concomitantly, the HM diet significantly reduced the expression of genes encoding tight junction proteins of the epithelial barrier (CHD1, CLDN2, CLDN3, and MLCK) in colon (Table 5). The ileal transcellular (HRP passage) permeability tended to be higher in HM-fed piglets, while no statistically significant difference was observed between HM- fed and IF-fed piglets for the colon (Table 4).

**TABLE 5:** Relative expression of differentially expressed genes (*p* < 0.1) in the ileum and the colon of HM- and IF-fed piglets (mean ± SEM).

Site	Function	Gene	Human milk	Infant formula	*P*-value
Ileum	Endocrine	DPPIV	1.04 ± 0.27	0.44 ± 0.11	0.047
		GHSR	1.08 ± 0.17	0.70 ± 0.20	0.043
		GLP1R	1.25 ± 0.14	0.41 ± 0.11	0.005
		NTS	1.05 ± 0.13	0.49 ± 0.12	0.028
	Immune system	IL10	1.02 ± 0.09	0.66 ± 0.06	0.003
	Tryptophan metabolism	IDO	1.15 ± 0.61	0.55 ± 0.49	0.037
		KYNU	1.07 ± 0.16	0.49 ± 0.18	0.005
		SERT	1.09 ± 0.22	1.65 ± 0.07	0.046
	Nutrient transporter	GLUT1	1.02 ± 0.09	0.78 ± 0.06	0.030
		PLA2G4	1.01 ± 0.32	0.72 ± 0.17	0.006
Colon	Barrier	Cdh1	1.04 ± 0.09	1.30 ± 0.08	0.046
		CLDN2	1.04 ± 0.18	1.44 ± 0.14	0.047
		CLDN3	1.04 ± 0.09	1.41 ± 0.11	0.007
		MLCK	1.04 ± 0.10	1.42 ± 0.12	0.029
	Endocrine	CCKBR	0.80 ± 0.29	0.25 ± 0.09	0.018
		CHGA	1.02 ± 0.12	1.74 ± 0.25	0.047
		IRS2	1.04 ± 0.13	0.66 ± 0.06	0.017
		NPY2R	0.77 ± 0.08	1.06 ± 0.21	0.028
		PCSK1	1.08 ± 0.17	1.75 ± 0.23	0.034
	Immune	BAFF	0.94 ± 0.24	0.27 ± 0.04	0.003
		CCL2	1.23 ± 0.33	0.50 ± 0.06	0.002
		CX3CL1	1.02 ± 0.09	0.72 ± 0.04	0.012
		ICAM1	1.06 ± 0.27	0.44 ± 0.06	0.002
		IL10	1.06 ± 0.16	0.29 ± 0.05	<0.001
		IL10Ra	1.11 ± 0.24	0.48 ± 0.06	0.003
		IL8	1.05 ± 0.29	0.18 ± 0.02	<0.001
		MYD88	1.04 ± 0.10	0.73 ± 0.02	0.005
		SOCS3	1.20 ± 0.27	0.44 ± 0.07	0.001
		TGFβ	1.15 ± 0.21	0.66 ± 0.08	0.023
		TLR2	1.02 ± 0.11	0.57 ± 0.04	0.002
		TLR4	1.09 ± 0.22	0.57 ± 0.07	0.029
		TNFa	1.09 ± 0.22	0.39 ± 0.12	0.007
		TNFaR1	1.00 ± 0.08	0.72 ± 0.06	0.010
	Tryptophan metabolism	AAAD	1.05 ± 0.15	1.31 ± 0.10	0.043
		AANAT	1.00 ± 0.28	0.49 ± 0.14	0.049
		IDO	0.46 ± 0.17	0.18 ± 0.07	0.038
		KYNU	1.02 ± 0.18	0.62 ± 0.03	0.016
		MAO	1.01 ± 0.08	1.84 ± 0.16	<0.001
		Tph1	1.07 ± 0.14	1.91 ± 0.29	0.018
	Nutrient transporter	FFAR2	1.22 ± 0.34	0.60 ± 0.27	0.031
		FFAR3	1.12 ± 0.19	0.50 ± 0.07	0.007
		GPR120	1.03 ± 0.10	0.76 ± 0.08	0.042
		MCT1	1.00 ± 0.12	1.62 ± 0.18	0.010
		MCT2	1.02 ± 0.08	1.29 ± 0.06	0.017
		MCT4	1.02 ± 0.09	0.68 ± 0.08	0.009
		NIACR1	0.59 ± 0.19	0.22 ± 0.06	0.019
		SLC38A5	1.02 ± 0.08	1.28 ± 0.07	0.019
		SLC6A19	1.17 ± 0.07	1.01 ± 0.20	<0.001
Liver	Tryptophan metabolism	TDO	1.04 ± 0.15	0.50 ± 0.05	0.006

### Enteroendocrine function

Ileal tissue GLP1 concentration was two times lower in HM-fed than in IF-fed piglets (Table 4). In contrast, no statistically significant diet effect was observed either for plasma or colonic tissue GLP1 concentration, nor on GLP1-secreting cell densities. The percentage of GLP1-secreting cells compared to all enteroendocrine cells tended to be higher in the ileal and colonic tissue of HM-fed piglets (Table 4). Concomitantly, a diet effect was found for the relative expression of genes involved in endocrine function (Table 5). Compared to IF-fed piglets, HM-fed piglets had upregulated ileal gene expression of GLP1R, GHSR, and NTS, and colonic gene expression of CCKBR, IRS2, and SOCS3, but downregulated colonic gene expression of CHGA, NPY2, and PCSK.

### Immune gene expression

Ileal IL10 and DPPIV gene expression was significantly higher and BAFF, CCL2, ICAM1, TGFβ2R, TLR2, and TOLLIP gene expression tended to be higher for HM-fed piglets (Table 5). Similarly, half of the analyzed genes involved in immune function were significantly upregulated in the colon of HM-fed piglets compared to the colon of IF-fed piglets. For instance, genes encoding anti-inflammatory immune response proteins such as IL10 and its receptor (IL10Ra), TGFβ, and BAFF, as well as genes encoding pro-inflammatory immune response proteins such as IL8, IL4, and TNFα were upregulated in the colon of HM-fed piglets. Likewise, TLR2, TLR4, and MYD88 genes were upregulated in the colon of HM-fed piglets.

### Tryptophan pathways and nutrient transporter gene expression

Among the selected genes involved in tryptophan metabolism, the expression of nine genes (three ileal and six colonic) were modulated by the dietary intervention, with upregulation of genes encoding proteins involved in the kynurenine pathway (KYNU, IDO, and AANAT) and downregulation of genes encoding proteins in the serotonin pathway (SERT, MAO, TPH1, and AAAD) in HM-fed

piglets compared to IF-fed piglets. In the liver, only the gene expression of TDO was significantly influenced by the diet, with upregulation in HM-fed piglets (Table 5).

Regarding nutrient transporters and digestion function, only GLUT1 and PLA2G4 gene expression was increased in the ileum of HM-fed piglets. In the colon, FFAR2 and FFAR3 genes were upregulated and MCT1 and MCT2 genes were downregulated in HM-fed piglets (Table 5).

### Brain gene expression

The proportion of differentially expressed genes between HM- and IF-fed piglets were high in the hypothalamus (38%) and striatum (18%) and low in the pre-frontal cortex (9%) and hippocampus (5%) (Table 6). All differentially expressed genes were downregulated in HM-fed piglets. For instance, in the hypothalamus, genes encoding tight junction proteins such as CDH2, CLDN12, OCLN, CTNNB1, or MARVELD2 were downregulated in HM- compared to IF-fed piglets. CDH2 and CLDN12 genes were also downregulated in the striatum of HM-fed piglets. In addition, the LSR gene was downregulated in HM-fed piglet striatum and pre-frontal cortex. The expression of genes involved in the endocrine function such as GLP1R, CCKBR, IRS1, and MME, was lower in HM hypothalamus. The pre-frontal cortex expression of INSR gene, the hippocampus expression of LEPR gene, and the striatum expression of CCKBR gene were lower in HM- vs. IF-fed piglets. Some genes encoding proteins of the immune function were significantly less expressed in hypothalamus (CX3CL1, TGFβ, and TLR4) and striatum (IL1bR) of HM-fed piglets. Genes implicated in the neurosynaptogenesis were significantly downregulated in the hypothalamus (BDNF, CSF1R, CYFIP2, FTO, and RANBP9) and the cortex (CSF1R and MOG) of HM-fed piglets. The expression of genes encoding neurotransmitters in the hypothalamus, GABBR1, GRIN2B, and NPY, was significantly impacted by diet, with a lower expression in the HM-fed piglets. Genes encoding SCFA receptors (FFAR2 and MCT1 in hypothalamus; FFAR2, FFAR3, MCT2, and MCT4 in striatum) were less expressed in HM-fed piglets. Finally, the expression of the 5HT2B gene in hypothalamus and hippocampus, and of that HTR1F and TPH2 genes in the striatum, was downregulated in HM-fed piglets.

**TABLE 6:** Relative expression of differentially expressed genes (*p* < 0.1) in four brain sites of HM- and IF-fed piglets (Mean ± SEM, significant *p*-values are in bold).

Site	Function	Gene	Human milk	Infant formula	*P*-value
Hypothalamus	Blood–brain barrier	CDH2	1.61 ± 0.63	2.48 ± 0.50	**0.039**
		CLDN12	1.40 ± 0.55	2.30 ± 0.78	**0.006**
		CTNNB1	1.51 ± 0.50	1.91 ± 0.35	**0.038**
		Marveld2	0.99 ± 0.16	3.07 ± 1.03	**0.005**
		OCLN	1.26 ± 0.42	1.81 ± 0.36	**0.004**
	Endocrine	CCKBR	2.12 ± 0.66	5.34 ± 0.71	**0.005**
		GLP1R	2.05 ± 1.29	2.96 ± 0.91	**0.010**
		IRS1	1.22 ± 0.33	2.27 ± 0.60	**0.001**
		MME	1.45 ± 0.43	5.46 ± 1.47	**0.004**
	Immune	CX3CL1	1.04 ± 0.22	1.67 ± 0.21	**0.009**
		TGFβ	1.13 ± 0.33	2.00 ± 0.53	**0.019**
		TLR4	1.56 ± 0.94	2.80 ± 0.94	**0.047**
	Neurosynaptogenesis	BDNF	1.55 ± 0.61	4.38 ± 1.00	**0.015**
		CSF1R	1.44 ± 0.52	2.68 ± 0.76	**0.004**
		CYFIP2	1.22 ± 0.28	2.40 ± 0.41	**0.002**
		DGL4	1.75 ± 0.79	3.07 ± 1.38	**0.011**
		FTO	1.11 ± 0.37	2.00 ± 0.46	**0.010**
		RANBP9	1.20 ± 0.35	2.04 ± 0.67	**0.005**
	Neurotransmitters	GABBR1	1.49 ± 0.52	2.55 ± 0.78	**0.008**
		GRIN2B	0.68 ± 0.26	1.03 ± 0.17	**0.007**
		NPY	1.68 ± 0.69	3.71 ± 0.88	**0.009**
	Tryptophan pathways	5HT2RB	1.52 ± 0.49	2.34 ± 0.54	0.053
	Nutrient transporter	FFAR2	1.74 ± 0.66	3.13 ± 0.82	**0.008**
		LRP1	1.22 ± 0.42	2.00 ± 0.53	**0.005**
		MCT1	1.21 ± 0.26	1.90 ± 0.40	**0.003**
Striatum	Blood–brain barrier	CDH2	0.99 ± 0.23	2.47 ± 0.53	**0.015**
		CLDN12	1.54 ± 0.30	2.73 ± 0.34	**0.003**
		LSR	0.98 ± 0.21	2.73 ± 0.74	**0.031**
	Endocrine	CCKBR	1.28 ± 0.30	3.35 ± 0.80	**0.017**
	Immune	IL1bR	1.30 ± 0.27	2.96 ± 0.97	**0.024**
	Tryptophan pathways	HTR1F	0.98 ± 0.34	2.01 ± 0.32	**0.040**
		TPH2	1.37 ± 0.34	4.27 ± 1.04	**0.009**
	Nutrient transporter	FFAR2	0.61 ± 0.13	2.92 ± 0.92	**0.007**
		FFAR3	0.83 ± 0.28	3.27 ± 0.99	**0.022**
		MCT2	0.99 ± 0.45	2.82 ± 0.53	**0.007**
		MCT4	0.96 ± 0.23	4.35 ± 1.52	**0.025**
Pre-fontal cortex	Blood–brain barrier	CLDN5	1.04 ± 0.13	1.70 ± 0.15	**0.004**
		LSR	1.08 ± 0.15	1.44 ± 0.13	**0.049**
	Endocrine	INSR	1.07 ± 0.16	1.61 ± 0.25	**0.036**
	Neurosynaptogenesis	CSF1R	1.02 ± 0.12	1.55 ± 0.17	**0.012**
		MOG	1.08 ± 0.16	2.00 ± 0.40	**0.019**
	Nutrient transporter	SLC27A4	1.04 ± 0.12	1.44 ± 0.11	**0.025**
Hippocampus	Endocrine	LEPR	0.83 ± 0.38	1.56 ± 0.29	**0.012**
	Tryptophan pathways	5HTR2B	0.73 ± 0.14	1.56 ± 0.51	**0.040**
	Nutrient transporter	LRP1	0.28 ± 0.01	1.04 ± 0.37	0.015

### Overall impact of the diet on the microbiota-gut-brain axis

**FIGURE 2:**
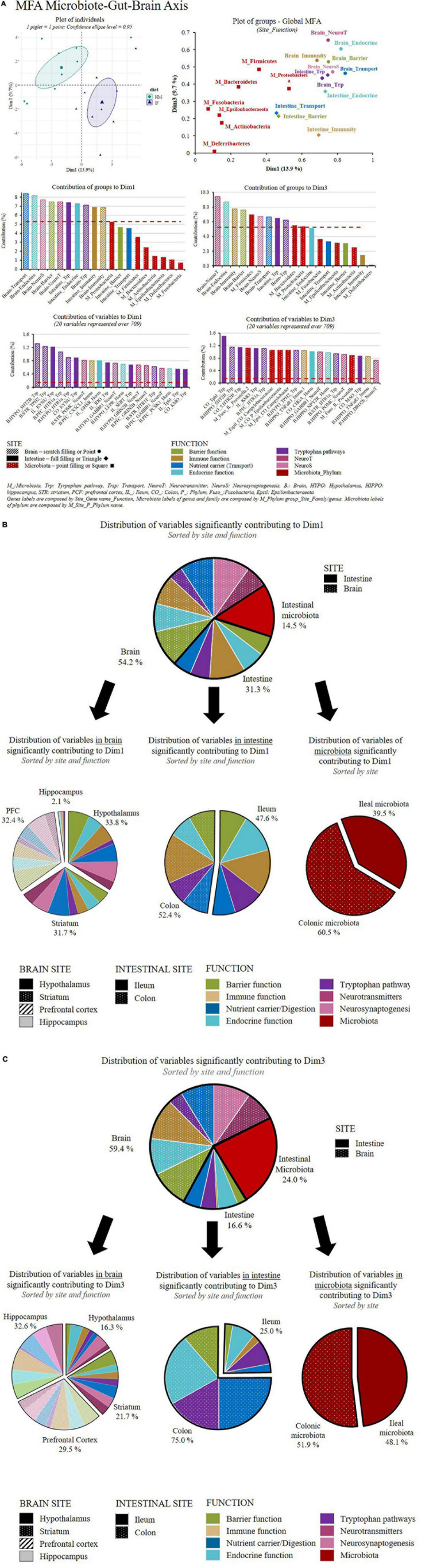
(**A)** Multiple factor analysis using microbiota, intestinal and brain variables analyzed in HM- and IF-fed piglets and grouped by site and function or phylum (*n* = 19 groups of variables). Red dot line: level of statistical significance corresponding to the inverse of the number of variable groups used in the analysis. **(B)** Distribution of variables contributing to Dim1 of the MFA as a percentage of variables which contribute for more than 0.141%. Selection criteria of 0.141% was calculated by dividing a base 100 by the total number of variables included in the MFA (n variables total = 709) and expressed as a percentage. PFC, prefrontal cortex. **(C)** Distribution of variables contributing to Dim3 of the MFA as a percentage of variables which contribute for more than 0.141%. Selection criteria of 0.141% was calculated by dividing a base 100 by the total number of variables included in the MFA (*n* variables total = 709) and expressed as a percentage.

The multifactorial analysis aimed to analyze the relationships between diet and groups of variables (*n* = 19), gathered by site (brain, intestine, and microbiota) and function (seven functions) or phylum (*n* = 7). HM and IF variables were discriminated on both dimensions 1 and 3 (25% of variance recovered, Figure 2) unlike that on dimension 1 and 2 representation (data not shown). The groups of variables contributing the most in the discrimination between HM- and IF-fed piglets on the first dimension implicated brain functions (neurosynaptogenesis, nutrient carrier, blood–brain barrier, endocrine function, neurotransmitter, and immune function). Those contributing the most in the separation of HM- and IF-fed piglets on the third dimension implicated microbiota (Firmicutes, Bacteroidetes, and Proteobacteria), brain functions (endocrine function, neurotransmitter, blood–brain barrier, and nutrient carrier), but also included variables representative of the intestinal functions (tryptophan metabolism, and endocrine function) (Figure 2A). Among all the variables included in this multifactorial analysis, 37% of them significantly contributed (% contribution > 0.14) to define the dimension 1, while 30% of them significantly contributed (% contribution > 0.14) to dimension 3. On a functional basis (Figures 2B,C), 15% (dimension 1) and 12% (dimension 3) of these significantly contributing variables were genes involved in brain and intestinal barrier function, respectively, 18% (dimension 1) and 11% (dimension 3) of them were genes involved in intestinal and brain immune function, 14% (dimension 1) and 14% (dimension 3) of them were genes involved in brain and intestinal endocrine functions, 14% (dimension 1) and 13% (dimension 3) of them were genes encoding nutrient carrier proteins in brain and intestine, and 9% (dimension 1) and 8% (dimension 3) of them were genes involved in intestinal and brain tryptophan pathways. An additional 6% (dimension 1) and 8% (dimension 3) of these variables were genes encoding for neurotransmitters in brain, and 10% (dimension 1) and 10% (dimension 3) were genes encoding for neurosynaptogenesis in brain. Finally, 15% (dimension 1) and up to 24% (dimension 3) of the variables significantly contributing to the definition of these dimensions were variables from the intestinal microbiota. Overall, this indicated that the diet affected the microbiota-gut-brain axis and their associated functions.

To provide a more thorough insight into the individual associations between variables, correlation analyses were performed between bacterial taxa (phyla, family, and genus levels) and ileal, colonic and brain tissue variables. Relative abundances of bacterial taxa were positively or negatively correlated (*p* < 0.05 and | *r*| > 0.7) with 10% of ileal genera correlated with 0.4% ileal variables analyzed, 33% of colonic genera with 1.0% colonic variables, and 48% of the gut genera with 0.6% cerebral variables.

**FIGURE 3:**
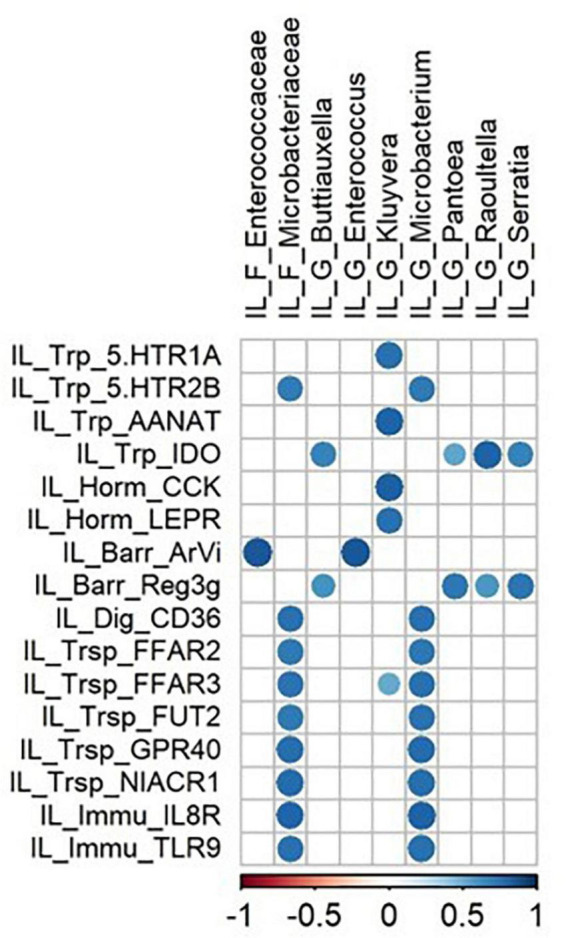
Correlations matrix between ileal variables (genes, morphometry data) and ileal microbiota (|*r*| ≥ 0.7, *P* < 0.05). IL, ileum; F, family; P, phylum; G, genera; ArVi, villous area; Trp, tryptophan pathways; Horm, endocrine function; Barr, barrier function; Dig, digestion function, Trsp, transport-nutrient carrier; Immu, immune function.

**FIGURE 4:**
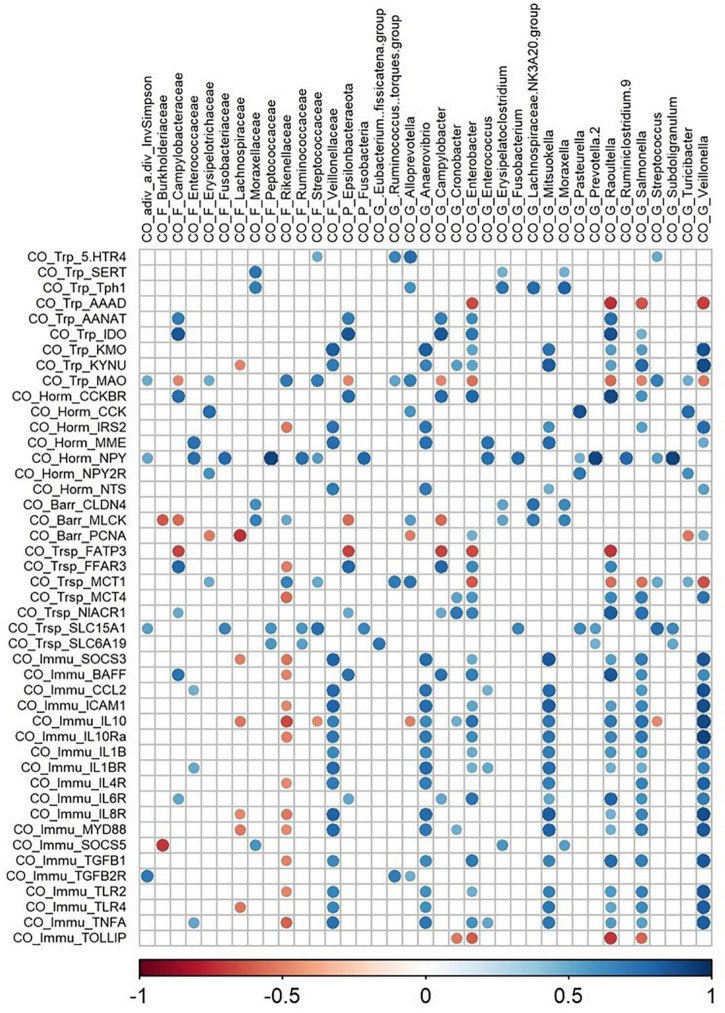
Correlations matrix between colonic relative gene expression and colonic microbiota (| *r*| ≥ 0.7, *P* < 0.05). CO, colon; F, family; P, phylum; G, genera; Trp, tryptophan pathways; Horm, endocrine function; Barr, barrier function; Trsp, transport-nutrient carrier; Immu, immune function.

At the ileal level, relative abundance of Microbacteriaceae and *Microbacterium* was positively correlated with genes related to immune function and nutrient carriers, and *Kluyvera* abundance was positively correlated to genes involved in endocrine function and tryptophan pathways (Figure 3). At the colonic level, *Anaerovibrio*, *Mitsuokella*, *Raoultella*, *Salmonella*, and *Veillonella* abundances were positively correlated and *Rikenellaceae abundance negatively correlated* with genes encoding for interleukins and their receptor, and toll-like receptors (TLR2 and TLR4) (Figure 4). Concerning the kynurenine pathway, *Anaerovibrio, Enterobacter, Mitsuokella, Raoultella, Salmonella*, and *Veillonella* were positively correlated with KMO and KYNU genes, *Campylobacter, Epsilonproteobacteria*, *Enterobacter*, and *Raoultella* were positively correlated with IDO and AANAT genes, whereas *Enterobacter*, *Raoultella, Salmonella*, and *Veillonella* were negatively correlated with AAAD (Figure 4). It is noticeable that NPY gene expression was positively correlated with several families and genera, specifically with *Prevotella* and *Subdoligranulum* genera.

**FIGURE 5:**
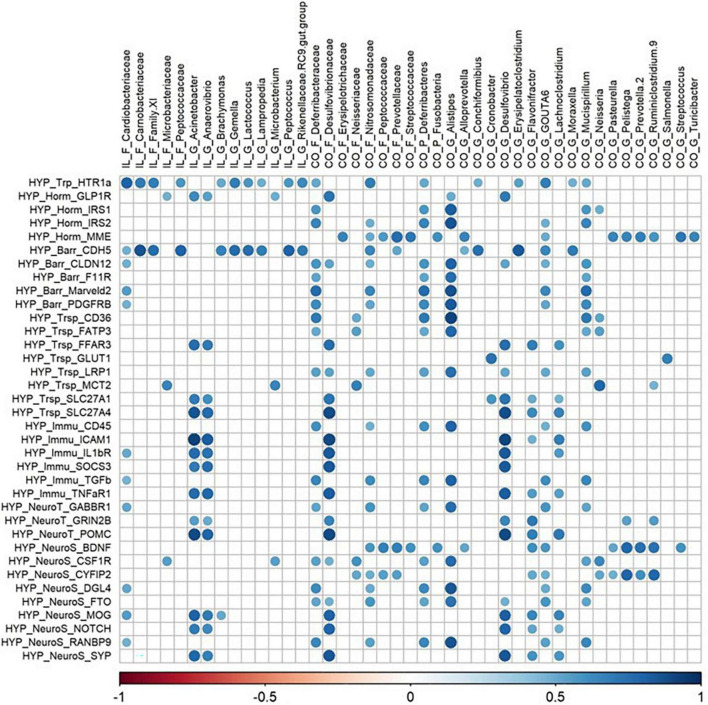
Correlations matrix between hypothalamic relative gene expression and ileal and colonic microbiota (| *r*| ≥ 0.7, *P* < 0.05). IL, ileum; CO, colon; F, family; P, phylum; G, genera; HYP, hypothalamus; Trp, tryptophan pathways; Horm, endocrine function; Barr, barrier function; Trsp, transport-nutrient carrier; Immu, immune function; NeuroT, neurotransmitters; NeuroS, neurosynaptogenesis.

**FIGURE 6:**
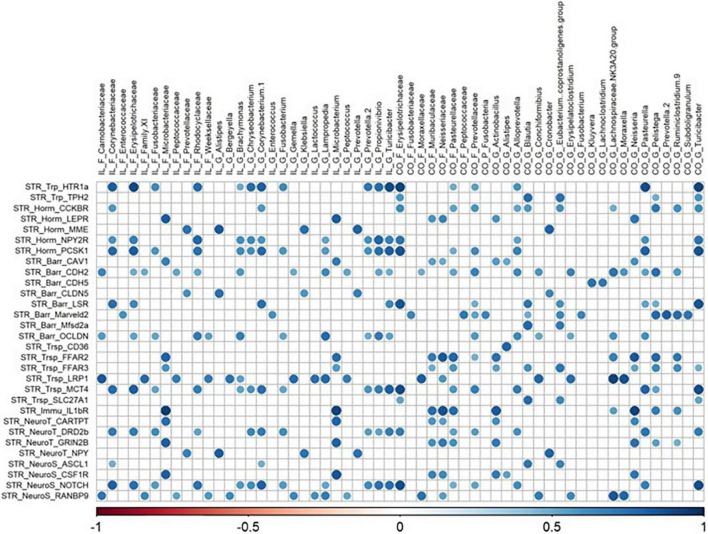
Correlations matrix between striatal relative gene expression and ileal and colonic microbiota (| *r*| ≥ 0.7, *P* < 0.05). IL, ileum; CO, colon; F, family; P, phylum; G, genera; STR, striatum; Trp, tryptophan pathways; Horm, endocrine function; Barr, barrier function; Trsp, transport-nutrient carrier; Immu, immune function; NeuroT, neurotransmitters; NeuroS, neurosynaptogenesis.

**FIGURE 7:**
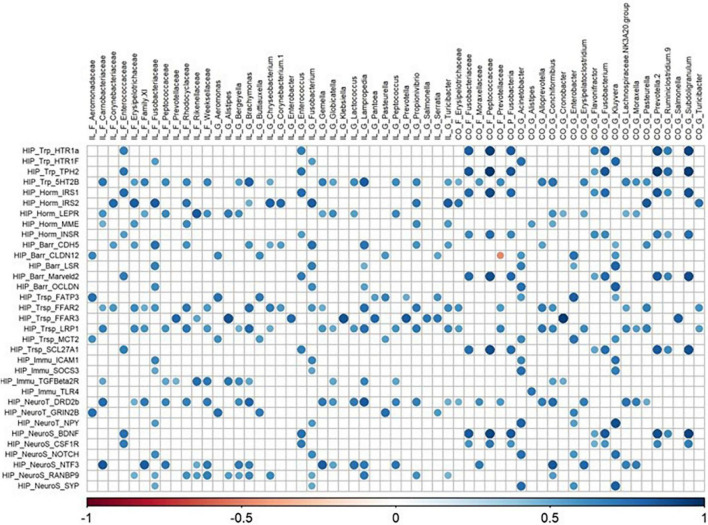
Correlations matrix between hippocampal relative gene expression and ileal and colonic microbiota (| *r*| ≥ 0.7, *P* < 0.05). IL, ileum; CO, colon; F, family; P, phylum; G, genera; HIP, hippocampus; Trp, tryptophan pathways; Horm, endocrine function; Barr, barrier function; Trsp, transport-nutrient carrier; Immu, immune function; NeuroT, neurotransmitters; NeuroS, neurosynaptogenesis.

**FIGURE 8:**
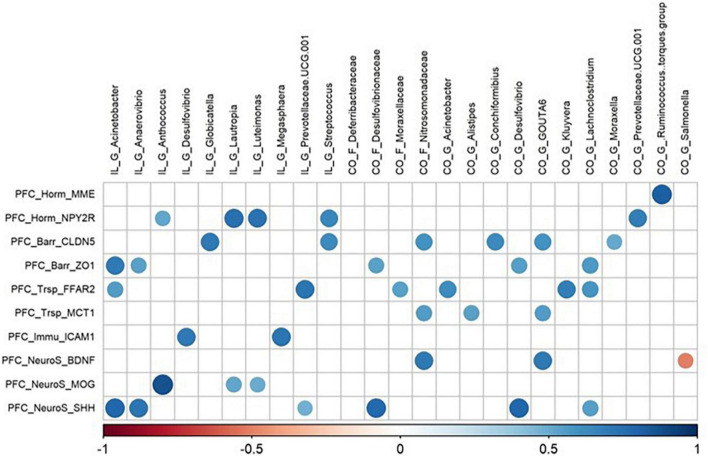
Correlations matrix between prefrontal cortex relative gene expression and ileal and colonic microbiota (| *r*| ≥ 0.7, *P* < 0.05). IL, ileum; CO, colon; F, family; P, phylum; G, genera; PFC, prefrontal-cortex; Trp, tryptophan pathways; Horm, endocrine function; Barr, barrier function; Trsp, transport-nutrient carrier; Immu, immune function; NeuroT, neurotransmitters; NeuroS, neurosynaptogenesis.

All matrix correlations between bacterial taxa (ileum and colon) and brain genes revealed positive correlations with brain site-dependent patterns. In the hypothalamus, correlations were found mainly with ileal *Acinetobacter* and *Anaerovibrio*, and colonic *Alistipes*, Deferribacteraceae, *Desulfovibrio*, *GOUTA6*, *Flavonifractor*, *Lachnoclostridium*, and *Mucispirillum* (Figure 5). All these taxa were correlated with the expression of 36 genes involved in functions distributed equally among all the cerebral functions studied. The expression of hypothalamic HTR1a gene involved in tryptophan pathways and CDH5 involved in blood–brain barrier were highly correlated with ileal taxa, while the expression of MME (hormonal regulation), and BDNF and CYFIP2 (neurosynaptogenesis) were highly correlated with colonic family and genera. The expression of 30 genes in the striatum and of 36 genes in the hippocampus, related to all functions studied, was correlated with both ileal and colonic microbiota (Figures 6, 7). It is notable that the gene encoding for the dopamine receptor (DRD2b) was correlated with several ileal and colonic taxa in the striatum and the hippocampus (Figures 6, 7). For the prefrontal cortex, the expression of only 10 genes was correlated to a few ileal and colonic taxa (Figure 8).

Moreover, several functions expressed in both intestinal and brain tissues were correlated one to each other, such as genes involved in immune, barrier and endocrine functions, tryptophan pathways, nutrient receptors and transporters or synaptogenesis (data not shown). In addition, it is remarkable that nine pairs of gene expressions highly correlated in the ileum and thirteen pairs of gene expression highly correlated in the colon were also significantly correlated in brain areas (Table 7).

**TABLE 7:** List of pairs of genes for which their expression was highly correlated (*r* > 0.7; *P* < 0.05) within the intestine and the brain.

Gene 1		Gene 2		Intestine		Brain			
Name	Function	Name	Function	*r* COLON	*r* ILEUM	*r* STR	*r* HYP	*r* HIP	*r* PFC
CCKBR	Endocrine	FFAR3	Nutrient carrier	0.75		0.79			
CTNNB1	Barrier	FATP3	Nutrient carrier	0.70				0.70	
CTNNB1	Barrier	SLC27A4	Nutrient carrier	0.76		0.85	0.85		0.87
FFAR2	Nutrient carrier	IL1BR	Immunity	0.71		0.90	0.75		
FFAR2	Nutrient carrier	IRS2	Endocrine	0.74			0.91		
ICAM1	Immunity	IL1BR	Immunity	0.82			0.87	0.71	
ICAM1	Immunity	IRS2	Endocrine	0.82		0.94			
ICAM1	Immunity	SOCS3	Immunity	0.98			0.82		
TGFβ	Immunity	IRS2	Endocrine	0.71			0.96		
TGFβ	Immunity	MCT4	Nutrient carrier	0.72			0.82	0.94	
TGFβ	Immunity	TLR4	Immunity	0.79			0.81		
TLR4	Immunity	IRS2	Endocrine	0.76			0.72		
TNFAR1	Immunity	MCT4	Nutrient carrier	0.75			0.71		
CTNNB1	Barrier	GLUT1	Nutrient carrier		0.74				0.84
CTNNB1	Barrier	IRS1	Endocrine		0.79				0.92
CTNNB1	Barrier	OCLN	Barrier		0.84		0.79		0.73
CTNNB1	Barrier	PDGFR	Barrier		0.86	0.73		0.81	
F11R	Barrier	OCLN	Barrier		0.91				0.72
F11R	Barrier	PDGFR	Barrier		0.73		0.83		
F11R	Barrier	SLC27A4	Nutrient carrier		0.89	0.71			
GLUT1	Nutrient carrier	IRS1	Endocrine		0.76	0.87		0.81	0.73
GLUT1	Nutrient carrier	PDGFR	Barrier		0.82				0.73

*r*, coefficient of correlation; STR, striatum; HYP, hypothalamus; HIP, hippocampus; PCF, prefrontal cortex.

## Discussion

For the first time, the impact of HM and IF diets on the collective microbiota-gut-brain axis in the piglet model has been demonstrated. Compared to IF, HM induced a different colonic and fecal microbiota profile, modulated intestinal gene expression, in particular those genes involved in the immune response, epithelial barrier, endocrine function, nutrients transporters and tryptophan metabolism. These results were in line with observed colonic physiological parameters, such as para-cellular permeability and the proportion of GLP-1 secreting cells. These diet-induced modifications were associated with modifications observed in the brain tissue expression of genes encoding the blood–brain barrier, endocrine function and SCFA receptors, mostly in the hypothalamic and striatal areas.

### The microbiota

The present study highlighted an impact of diet on the colonic and fecal microbiota composition, such as previously reported in infants over the first months of life (30, 58). Consistent with results reported for 0 to 6 month-old breastfed infants and HM-fed piglets (26, 27, 59), the present study also showed that the fecal and colonic α-diversity decreased in HM-fed piglets compared to IF counterparts after a 5-day dietary intervention. More specifically, fecal and colonic Bacteroidetes and Fusobacteria phyla were significantly here decreased in the HM-fed piglets, such as was observed in infants (26) and piglets (59). Among differentially abundant bacterial families, the lower abundance of Campylobacteraceae and the higher abundance of Prevotellaceae were previously observed in the colon of HM-fed piglets (60). Several dietary factors may explain the difference observed in the present study. Particularly, HM oligosaccharides likely contributed to the higher abundance of *Bifidobacterium*, *Lactobacillus*, and *Bacteroides* in HM feces (61–63) as well as the high urea content in HM, which can be used as a substrate by *Bifidobacterium* (64). In addition, the quality of the dietary lipids (plant in IF vs. animal in HM) may have also contributed to the modulation of the intestinal microbiota composition, such as demonstrated previously (1). In the present study, the statistically significant correlations found between SCFA transporters or receptors and Bacteroidetes, Firmicutes and Proteobacteria phyla in the ileum and colon are consistent with an effect of the origin (plant based vs. milk fat) of infant formula lipids (1, 51, 65, 66). In contrast, no difference in ileal microbial diversity was observed between HM- and IF-fed piglets, indicating that the most decipherable impact of the dietary treatments on the microbiota occurred in the large intestine where non-digestible molecules, especially HM oligosaccharides present in HM and urea are fermented by commensal bacteria (64, 67, 68).

Despite the pasteurization applied to the HM pool, the present study conducted to similar results as those found in the literature for piglets and infants fed with fresh HM in comparison with an IF. The present comparison highlights the prebiotic and postbiotic role of HM in infant nutrition rather than its probiotic role. Moreover, the present observations are in compliance with previous work studying the effect of pasteurization on HM health outcomes (69). Therefore, changes in microbiota and gut-brain axis parameters are assumed to mainly result from the pasteurized milk feeding.

### Intestinal physiology

The diet-induced changes in intestinal gene expression were greater in the colon (38% of total targeted genes) than in the ileum (8%), such as observed for the microbiota.

#### Immune and barrier functions

A remarkable observation in the present study was the higher colonic expression of genes encoding pro- but also anti-inflammatory cytokines and their receptors in HM- compared to IF-fed piglets. Such an observed HM-induced boost of the mucosal immune system agrees with data reported in breastfed infants (70–72). For instance, several studies have reported a higher fecal calprotectin content in HM-fed infants over the first weeks of life compared to IF-fed ones. Calprotectin has been considered as a valuable marker of intestinal mucosa inflammatory infiltration by neutrophils in response to early bacterial colonization (6–9). Moreover, the importance of the Firmicutes-induced pre-weaning peak of intestinal inflammatory markers was demonstrated in rodents as an essential phase for both immune ontogeny and regulation of susceptibilities to immunopathologies later in life (32). Positive correlations between fecal calprotectin excretion and colonization by *Staphylococcus* and *Clostridium* (Firmicutes phylum) sustained the role of bacteria in the maturation of the intestinal immune system (73). Accordingly, our data support the relationship between microbiota and the mucosal immune system maturation. Veillonellaceae (Firmicutes) were more abundant in the colon and feces of HM-fed piglets. Several significant positive correlations were observed between *Anaerovibrio*, *Mitsuokella*, and *Veillonella* genera belonging to this family and genes encoding anti- and pro-inflammatory cytokines (IL10, IL10Ra, SOCS3, CCL2, IL1bR, IL8, and TNFα) and cellular signaling (ICAM1 and MYD88). Other immunomodulatory factors, such as lactoferrin or other minor proteins present in HM (74) but not in IF, are likely to have contributed to the immune system boost.

The positive correlations found here between Lachnospiraceae (Firmicutes) and Moraxellaceae (Proteobacteria) and genes encoding barrier function (CLDN4 and MLCK) illustrate the relationship between the microbiota and intestinal barrier function. Colonic genes encoding tight junction proteins that sustain epithelial barrier integrity (75) were less expressed in HM- than in IF-fed piglets, in line with the observed increased colonic epithelial paracellular permeability. It is noteworthy that the measured permeability values of paracellular and transcellular permeabilities were in the physiological range of values reported in sow milk-fed piglets (52). The results are in agreement with the study of Lee et al. (10), who showed a higher permeability in breastfed infants, but do not corroborate other studies that reported no change or a reduction of the total gut permeability in breastfed infants compared to IF-fed infants (6, 11, 76, 77) or a reduced expression of genes encoding tight junction proteins in HM-fed piglets (78) when determined at a specific time point. When paracellular permeability was measured in the first month of age in suckling piglets, a three- to ten-fold increase was observed in the ileum with a smaller increase in the colon (52, 76). Therefore, feeding with HM may promote an age-induced increase of intestinal permeability that coincides with the evolution of mucosal immune cells present in the intestine of young pigs. A high intestinal permeability may allow for an increased passage of molecules through the epithelium, thereby influencing the maturation of the immune system and promoting the acquisition of tolerance against indigenous bacterial and dietary antigens (76). It is well known that pig neonates are skewed toward a Th2 profile and the balance between Th1 and Th2 responses develops progressively during the first 3–4 weeks of age. An enhancement of the intestinal immune system development observed in suckling piglets whose mothers were fed a prebiotic-supplemented diet (50), was shown to have further beneficial consequences by strengthening gut defenses and vaccine immune response post-weaning (79). Therefore, the higher ileal and colonic permeability, associated with an acceleration of the intestinal immune system maturation and changes in microbiota composition reported here in HM-fed piglets may constitute a key component of the lifelong breastfeeding health benefits.

#### Endocrine function

No dietary effect was observed on the concentrations of colonic and fecal SCFAs and GLP1, and GLP1R genes were not significantly correlated with bacterial taxa, suggesting that microbiota and bacterial metabolites did not play a key role in the modulation of GLP1 release in our study. Surprisingly, the ileal GLP1 content was two-fold lower in HM-fed piglets. Therefore, the reduced GLP1 content may result in a lower insulin secretion (80), corroborating the few data in the literature pointing to a lower insulinemic response in breastfed infants compared to IF fed infants (81). A similar effect of “pancreatic savings” has been previously observed in piglets supplemented with prebiotics or probiotics (50, 82). In addition to its effects on insulin secretion, GLP1 also takes part in central regulation of food intake, emotional eating and mood (83). Further investigations are needed to characterize a diet effect on central regulation of emotional eating and mood.

#### Tryptophan metabolism

The diet contributed significantly to the modulation of the tryptophan metabolism pathways. The differential ileal and colonic expression of genes involved in tryptophan metabolism was in favor of the kynurenine pathway in HM-fed piglets and in favor of the serotonin pathway in IF-fed piglets in agreement with Brink et al. (59), who reported a significant increase of kynurenic acid in HM-fed piglets. In the present study, positive correlations were found between the expression of intestinal (ileal or colonic) IDO, KMO, and KYNU genes and pro-inflammatory genes (BAFF, CCL2, IL1B, IL6R, IL23A, IL8, and TNFα) in agreement with the inflammation-induced kynurenine pathway (33, 84).

There were also positive correlations in the colon between KMO and KYNU gene expression and *Anaerovibrio*, *Mitsuokella*, and *Veillonella* genera (Veillonellaceae family) and in the ileum between 5-HTR1A, AAANT, TPH1, and Enterobacteriaceae and Corynebacteriaceae families and *Propionivibrio*, which is in agreement with the known influence of gut bacteria in serotonin and kynurenine pathway induction (36, 85). Positive correlations between TPH1 gene expression and Erysipelotrichaceae and Lachnospiraceae families corroborate that observed in 6-month-old breastfed infants (86). Moreover, *Lactobacillus* and *Bacteroides* were more abundant in HM-fed piglets, which could influence indole metabolism, such as previously reported (33). Indole-3-propionic acid has been previously shown to be less abundant in HM-fed piglets (60). Further investigation of the specific HM components shaping the bacterial metabolomic profile is warranted. Despite a similar tryptophan content in HM and IF, the protein containing tryptophan differed, particularly regarding α-lactalbumin, present in HM and in much smaller quantity in IF. In addition, the protein structure differed in the diet, being altered in IF due to the heat treatment applied for IF production. Whether the tryptophan release differs during HM and IF digestion remains unknown.

### Brain gene expression

Interestingly, HM and IF diets induced differential expression profiles of genes encoding blood–brain barrier, endocrine and immune functions and neurosynaptogenesis in the four studied brain areas, and more particularly in the hypothalamus and striatum.

It is acknowledged that microbiota can modulate brain function (18). Accordingly, at a family level, Lachnospiraceae and Enterobacteriaceae were positively correlated with genes encoding SCFA’s and monocarboxylate transporters in the brain and blood-brain barrier function (87, 88). It is noteworthy that the genus *Alistipes* (Rikenellaceae) was correlated with numerous genes encoding blood-brain barrier, immune, endocrine and neurosynaptogenesis functions, particularly in the hypothalamus. This genus, generally considered as a commensal of human gut microbiota, ferments undigested proteins that escape digestion in the small intestine (89, 90), suggesting that a different amount of undigested or partially digested protein between HF and IF may reach the colon. Although there is contrasting evidence for on the critical role *Alistipes* plays in inflammation, gastrointestinal and behavior disorders (37, 91–93), the present results suggest that this genus may be a key actor in the microbiota-brain axis.

### Microbiota-gut-brain axis: Global overview

Overall, the multifactorial analysis indicated that the brain variables, representative of most of the functions studied, the intestinal variables (mainly representative of the endocrine function and tryptophan metabolism), and the microbiota variables contributed to the differences observed for the HM-and IF-fed piglets. It illustrates the complexity of the relationships between the intestine and brain areas in interaction with the dietary treatment. Correlation analyses highlighted specific families (Veillonellaceae, Enterobacteriaceae, Lachnospiraceae, Rikenellaceae, and Prevotellaceae) whose relative abundance was correlated with several ileal, colonic and brain variables involved in different functions such as tryptophan metabolism, endocrine and barrier functions. It is remarkable that all statistically significant ileal and colonic taxa correlations with brain parameters were positive correlations. Moreover, specific ileal bacteria were correlated to the expression of similar genes in both ileum and brain content. These correlations concerned *Corynebacterium* and 5HTR1a, *Microbacterium* and LEPR, and *Actinotignum* and FFAR3. These data suggest that microbiota may act on similar functions (tryptophan metabolism, endocrine, and nutrient transport) shared on the gut-brain axis and may be key components of specific pathways. However, due to the limited data available in the literature, it is not possible to conclude on the reason of these correlations.

Finally, it should be born in mind that pasteurized HM had to be used for the piglet feeding as it was not ethically and technically possible to use such a large volume of fresh HM. It has been demonstrated that Holder pasteurization (30 min, 62.5°C) induces some protein denaturation, particularly for lactoferrin or for other bioactive and heat-sensitive proteins such as immunoglobulins or bile salt-stimulated lipase (69). This can in turn modulate their digestion (94, 95). However, beneficial health outcomes of pasteurized HM are still acknowledged (69). Using fresh HM during the entire experimentation may have enhanced the observed differences.

## Conclusion

The microbiota-gut-brain axis was modulated by the diet, with the microbiota probably playing an interface role between the diet and the host, especially in the colon. Particularly, the HM-associated microbiota profile likely improved the maturation of the intestinal epithelial barrier, immune system and endocrine functions, and modulated intestinal and cerebral tryptophan metabolism as well as several other cerebral functions in the early period of life. Undigestible nutrients such as HM oligosaccharides and urea may have contributed to the different microbiota profile. Other bioactive components of HM may also have likely contributed to the observed effects, either directly or through the microbiota. Further investigations focused on the dietary components of the IF would be useful.

## Data availability statement

The datasets presented in this study can be found in online repositories. The names of the repository/repositories and accession number(s) can be found below: https://entrepot.recherche.data.gouv.fr/dataset.xhtml?persistentId=doi:10.57745/5FHAYQ and https://doi.org/10.57745/5FHAYQ.

## Ethics statement

The studies involving human participants were reviewed and approved by the Institutional Review Board of South Mediterranean V (no. 19.12.12.65653). The patients/participants provided their written informed consent to participate in this study. This animal study was reviewed and approved by Ethics Committees of CREEA (Rennes Committee of Ethics in Animal Experimentation) France’s Ministry of Higher Education and Research approved the protocol (authorization #2020020610329770).

## Author contributions

DD, AD, and IL contributed to the conception and design of the study. EC, AlB, AmB, YL-G, PD, VR, GR, AC, AD, and IL collected the data. EC and AlB organized the database. EC, AlB, IL, and AD performed statistical analysis. EC wrote the first draft of the manuscript. AD, IL, and EC wrote section of the manuscript. PM, CM, DD, AD, and IL contributed to the funding of the study. All authors contributed to manuscript revision, read, and approved the submitted version.
