# Supplementary material for: Infant nutrition affects the microbiota-gut-brain axis: Comparison of human milk vs. infant formula feeding in the piglet model
Source: Front Nutr. 2022 Sep 21;9:976042. doi: 10.3389/fnut.2022.976042 (PMC9532976; doi:10.3389/fnut.2022.976042)
Supplement: Supplementary file 1 [file Data_Sheet_1.PDF]

Supplemental information

SUPPLEMENTARY TABLE 1 Nutritional composition of the full fat bovine milk powder used as the adaptation diet

Component	g /100 g full fat bovine milk powder	g/L adaptation diet
Lactose	38	54.71
Lipids	26	37.44
Proteins	26	37.44
Minerals	6	8.64
Added vitamins and minerals	-	1.79

Supplementary table 2 Gene list of the Porcine Gut Smartchip

General function	Sub-function	Gene name	Protein name
Barrier	Permeability	CDH1	Cadherin 1
		CLDN2	Claudin 2
		CLDN3	Claudin 3
		CLDN4	Claudin 4
		CLDN7	Claudin 7
		CTNNB1	β-catenin
		F11R	Junctional adhesion molecule-A (JAM-A)
		KLF4	Kruppel Like Factor 4
		MARVELD2	Tricellulin
		MLCK	Myosin light-chain kinase
		OCLN	Occludin
		PDGFRB	Platelet-derived growth factor receptor B
		ZO1	Zonulin 1
	Defense	BD2	Beta defensin 2
		IAP	Intestinal Alkaline Phosphatase
		LYZ	Lysozyme (1 ou P)
		MUC1	Mucin 1
		MUC2	Mucin 2
		PCNA	Proliferating Cell Nuclear Antigen
		REG3G	Regenerating islet-derived 3 gamma
		TFF3	Trefoil factor 3
Endocrine	Endocrine	CCK	Cholecystokinin
		CCKBR	Cholecystokinin B Receptor
		CHGA	Chromogranin A
		DPPIV	Dipeptidyl peptidase IV
		GHSR	Growth Hormone Secretagogue Receptor
		GLP1	Glucagon Like Peptide 1
		GLP1R	Glucagon Like Peptide 1 receptor
		INSR	Insulin receptor
		IRS1	Insulin Receptor Substrate 1
		IRS2	Insulin Receptor Substrate 2
		LEPR	Leptin receptor
		MME	Membrane Metalloendopeptidase
		NPY	Neuropeptide Y
		NPY1R	Neuropeptide Y Receptor Y1
		NPY2R	Neuropeptide Y Receptor Y2
		NTS	Neurotensin
		PCSK1	Proprotein convertase subtilisin/kexin type 1 inhibitor
		PYY	Peptide YY (Peptide Tyrosine Tyrosine)
		SSTR5	Somatostatin Receptor Type 5
Immune system	Anti-inflammatory	BAFF	B-cell activating factor
		IL10	Interleukin 10
		IL10RA	Interleukin 10 Receptor Subunit Alpha
		SOCS3	Suppressor Of Cytokine Signaling 3
		SOCS5	Suppressor Of Cytokine Signaling 5
		TGFB	Transforming Growth Factor Beta
		TGFB2R	Transforming Growth Factor Beta 2 receptor
	Receptor/cellular signaling	ICAM1	Intercellular Adhesion Molecule 1
		MYD88	Myeloid Differentiation Primary Response Gene (88)
		PIGR	Polymeric Immunoglobulin Receptor
		TLR2	Toll-like receptor 2
		TLR4	Toll-like receptor 4
		TLR5	Toll-like receptor 5
		TLR9	Toll-like receptor 9
		TOLLIP	Toll interacting protein
	Pro-inflammatory	CCL2	C-C Motif Chemokine Ligand 2
		CCL20	C-C Motif Chemokine Ligand 20
		CX3CL1	C-X3-C Motif chemokine Ligand 1 (Fractalkine)
		IFNG	Interferon gamma
		IL1B	Interleukin 1-Beta

		IL1BR	Interleukin 1-Beta receptor
		IL23A	Interleukin 23A
		IL4R	Interleukin 4 receptor
		IL6R	Interleukin 6 receptor
		IL8	Interleukin 8
		IL8R	Interleukin 8 receptor
		TNFA	Tumor necrosis factor alpha
		TNFAR1	Tumor necrosis factor alpha receptor 1
Nutrient transporter	Amino acid transporter	SLC15A1	Peptide transporter 1
		SLC1A1	Sodium-Dependent Glutamate/Aspartate Transporter 3
		SLC38A2	Sodium-Coupled Neutral Amino Acid Transporter 2
		SLC38A5	Sodium-coupled neutral amino acid transporter 5
		SLC6A19	Sodium-Dependent Neutral Amino Acid Transporter B0
	Carbohydrate transporter	GLUT1	Glucose Transporter Type 1
		GLUT2	Glucose Transporter Type 2
		GLUT5	Fructose Transporter Type 5
		SGLT1	Sodium/glucose Cotransporter 1
	Fatty acid transporter	CD36	Fatty acid translocase/CD36 (FAT/CD36)
		FATP3	Fatty acid transporter 3
		GPR120 (FFAR4)	Free fatty acid receptor 4
		GPR40 (FFAR1)	Free fatty acid receptor 1
		SLC27A1 (FATP1)	Long-chain Fatty Acid Transport Protein 1
		SLC27A4 (FATP4)	Long-chain Fatty Acid Transport Protein 4
	SCFA receptor/transporter	FFAR2	Free fatty acid receptor 2
		FFAR3	Free fatty acid receptor 3
		MCT1	Monocarboxylate Transporter 1
		MCT2	Monocarboxylate Transporter 2
		MCT4	Monocarboxylate Transporter 4
		NIACR1	Niacin receptor 1
	Digestion	FUT2	Fucosyltransférase 2
		LPH	Lactase Phlorizine hydrolase
		PLA2G4	Phospholipase A2 Group IVA
		SI	Sucrase-isomaltase
Tryptophan pathways	Kynurenine pathway	IDO	Indoleamine 2,3-Dioxygenase 1
		KMO	Kinurenine 3-monooxygenase
		KYAT	Kinurenine aminotransferase
		KYNU	Kynureninase
	Serotonin pathway	5-HTR1A	5-Hydroxytryptamine receptor 1A
		5-HTR2B	5-Hydroxytryptamine receptor 2B
		5-HTR4	5-Hydroxytryptamine receptor 4
		AAAD	Aromatic amino acid decarboxylase
		AANAT	Aralkylamine N-Acetyltransferase
		MAO	Monoamine Oxydase
		SERT	Serotonin transporter
		TPH1	Tryptophan hydroxylase 1
	Tryptophan pathway	AHR	Aryl Hydrocarbon Receptor
Housekeeping gene	Housekeeping gene	ACTB	Actin beta
		ALDOA	Aldolase, Fructose-Bisphosphate A
		B2M	Beta-2-Microglobulin
		GAPDH	Glyceraldehyde-3-Phosphate Dehydrogenase
		HPTR1	Hypoxanthine Phosphoribosyltransferase 1
		PGK1	Phosphoglycerate Kinase 1
		POLR2G	RNA Polymerase II Subunit G
		PPIA	Peptidylprolyl Isomerase A
		RPL4	Ribosomal Protein L4
		RPLP	Ribosomal Protein Lateral Stalk Subunit P
		SDHA	Succinate Dehydrogenase Complex Flavoprotein Subunit A
		TOP2B	DNA Topoisomerase II Beta
		YWHAZ	Tyrosine 3-Monooxygenase/Tryptophan 5-monooxygenase Activation Protein Zeta

Supplementary table 3 Gene list of the Porcine Brain Smartchip

General function	Gene name	Protein name
Blood-Brain-Barrier	CAV1	Caveolin 1
	CDH2	Cadherin 2
	CDH5	Cadherin 5
	CLDN12	Claudin 12
	CLDN5	Claudin 5
	CTNNB1	β-catenin
	F11R	Junctional adhesion molecule-A (JAM-A)
	LAMC1	Laminin Subunit Gamma 1
	LSR	Lipolysis Stimulated Lipoprotein Receptor
	MARVELD2	tricellulin
	MFSD2A	Sodium-Dependent Lysophosphatidylcholine Symporter 1
	OCLN	Occludin
	PDGFRB	Platelet-derived growth factor receptor B
	ZO1	Zonulin 1
GIT hormones and receptors	CB1	Cannabinoid Receptor 1
	CCKBR	Cholecystokinin B Receptor
	GLP1R	Glucagon Like Peptide receptor 1
	INSR	Insulin Receptor
	IRS1	Insulin Receptor Substrate 1
	IRS2	Insulin Receptor Substrate 2
	LEPR	Leptin receptor
	MME	Membrane Metalloendopeptidase
	NPY2R	Neuropeptide Y Receptor Y2
	PCSK1	Proprotein convertase subtilisin/kexin type 1 inhibitor
Immune system	AIF1	Allograft Inflammatory Factor 1
	CD45	Protein Tyrosine Phosphatase Receptor Type C
	CX3CL1	C-X3-C Motif chemokine Ligand 1 (Fractalkine)
	ICAM1	Intercellular Adhesion Molecule 1
	IL1BR	Interleukin 1 Beta receptor
	SOCS3	Suppressor Of Cytokine Signaling 3
	TGFB	Transforming Growth Factor Beta
	TGFB2R	Transforming Growth Factor Beta 2 receptor
	TLR4	Toll-like receptor 4
	TNFAR1	Tumor necrosis factor alpha receptor 1
neurosynaptogenesis	ASCL1	Achaete-Scute Family BHLH Transcription Factor 1
	BDNF	Brain Derived Neurotrophic Factor
	CSF1R	Colony Stimulating Factor 1 Receptor
	CYFIP2	Cytoplasmic FMR1 interacting protein 2
	DGL4	Postsynaptic density protein-95 (PSD95) ou DGL4 ou SAP-90
	FTO	FTO Alpha-Ketoglutarate Dependent Dioxygenase
	MOG	Myelin Oligodendrocyte Glycoprotein
	NOTCH	Notch protein
	NTF3	Neurotrophin 3
	RANBP9	Ran-Binding Protein 9
	SHH	Sonic Hedgehog signaling molecule
	SYP	Synaptophysin
neuro-transmitter	AGRP	Agouti Related Neuropeptide
	CARTPT	Cocaine- And Amphetamine-Regulated Transcript Protein
	DRD2B	Dopamine Receptor D2b
	GABRA1	Gamma-Aminobutyric Acid Type A Receptor Subunit Alpha1
	GABRB1	Gamma-Aminobutyric Acid Type B Receptor Subunit Alpha1
	GRIN2B	Glutamate Receptor, Ionotropic, N-Methyl D-Aspartate 2B
	NPY	Neuropeptide Y
	POMC	Proopiomelanocortin
Nutrient transporter	CD36	Fatty acid translocase/CD36 (FAT/CD36)
	FATP3	Fatty acid transporter 3
	FFAR2	Free fatty acid receptor 2
	FFAR3	Free fatty acid receptor 3
	GLUT1	Glucose Transporter Type 1
	LRP1	LDL Receptor Related Protein 1
	MCT1	Monocarboxylate Transporter 1
	MCT2	Monocarboxylate Transporter 2
	MCT4	Monocarboxylate Transporter 4
	SLC27A1	Long-chain Fatty Acid Transport Protein 1

	SLC27A4	Long-chain Fatty Acid Transport Protein 4
Tryptophan metabolism	5HTR2B	5-Hydroxytryptamine (Serotonin) receptor 2B
	HTR1A	5-Hydroxytryptamine Receptor 1A
	HTR1F	5-Hydroxytryptamine Receptor 1F
	TPH2	Tryptophan hydroxylase 2
Housekeeping gene	ACTB	Actin beta
	ALDOA	Aldolase, Fructose-Bisphosphate A
	B2M	Beta-2-Microglobulin
	HPTR1	Hypoxanthine Phosphoribosyltransferase 1
	PGK1	Phosphoglycerate Kinase 1
	PPIA	Peptidylprolyl Isomerase A
	RPL4	Ribosomal Protein L4
	RPLP	Ribosomal Protein Lateral Stalk Subunit P
	SDHA	Succinate Dehydrogenase Complex Flavoprotein Subunit A
	YWHAZ	Tyrosine 3-Monooxygenase/Tryptophan 5-Monooxygenase Activation Protein Zeta

Supplementary table 4 Fecal and colonic SCFA composition before and after distribution of specific diets (mean ± sem, mmol/kg digesta)

	Adaptation diet period	Specific diet period			
	Feces	Feces		Colon	
	All piglets	HM group	IF group	HM group	IF group
Acetate (C2)	20.68 ± 3.34	18.87 ± 2.48	21.88 ± 9.20	57.29 ± 7.71	75.00 ± 8.11
Propionate (C3)	5.79 ± 1.36	6.97 ± 1.01	8.15 ± 3.51	22.09 ± 2.95	30.90 ± 6.28
Isobutyrate (IC4)	0.58 ± 0.18	0.10 ± 0.08	0.16 ± 0.08	0.23 ± 0.18	0.00 ± 0.00
Butyrate (C4)	1.81 ± 0.39	1.24 ± 0.31	1.83 ± 0.60	4.85 ± 1.41	4.57 ± 1.81
Isovalerate (IC5)	1.05 ± 0.30	0.58 ± 0.26	0.24 ± 0.12	0.40 ± 0.27	0.51 ± 0.23
Valerate (C5)	0.82 ± 0.40	1.34 ± 0.50	0.35 ± 0.08	2.67 ± 0.97	0.60 ± 0.12
TOTAL SCFA	30.74 ± 5.56	29.10 ± 3.74	32.58 ± 13.27	87.53 ± 10.97	111.59 ± 14.59

SCFAs were analyzed by High Performance Liquid Chromatography (HPLC, Ultimate 3000, Thermo Fisher Scientific 91941 Courtaboeuf). Quantification was performed with an external calibration using acetic acid (PanReac, Lyon, France), propanoic, 2-methylpropanoic, butanoic, 3-methylbutanoic and pentanoic acids (Merck, St. Quentin Fallavier, France) as standards. Statistical analyses were processed as explained in part 2.10.2 Unidimensional analysis, non-significant differences were obtained at each period and in each site.
